# Chromatin accessibility differences between alpha, beta, and delta cells identifies common and cell type-specific enhancers

**DOI:** 10.1186/s12864-023-09293-6

**Published:** 2023-04-17

**Authors:** Alex M. Mawla, Talitha van der Meulen, Mark O. Huising

**Affiliations:** 1 Department of Neurobiology, Physiology & Behavior, College of Biological Sciences, University of California, One Shields Avenue, Davis, CA 95616 USA; 2 Department of Physiology and Membrane Biology, School of Medicine, University of California, One Shields Avenue, Davis, CA 95616 USA

**Keywords:** Epigenomics, Epigenetics, Diabetes, Chromatin, Enhancers, ATAC-Seq

## Abstract

**Background:**

High throughput sequencing has enabled the interrogation of the transcriptomic landscape of glucagon-secreting alpha cells, insulin-secreting beta cells, and somatostatin-secreting delta cells. These approaches have furthered our understanding of expression patterns that define healthy or diseased islet cell types and helped explicate some of the intricacies between major islet cell crosstalk and glucose regulation. All three endocrine cell types derive from a common pancreatic progenitor, yet alpha and beta cells have partially opposing functions, and delta cells modulate and control insulin and glucagon release. While gene expression signatures that define and maintain cellular identity have been widely explored, the underlying epigenetic components are incompletely characterized and understood. However, chromatin accessibility and remodeling is a dynamic attribute that plays a critical role to determine and maintain cellular identity.

**Results:**

Here, we compare and contrast the chromatin landscape between mouse alpha, beta, and delta cells using ATAC-Seq to evaluate the significant differences in chromatin accessibility. The similarities and differences in chromatin accessibility between these related islet endocrine cells help define their fate in support of their distinct functional roles. We identify patterns that suggest that both alpha and delta cells are poised, but repressed, from becoming beta-like. We also identify patterns in differentially enriched chromatin that have transcription factor motifs preferentially associated with different regions of the genome. Finally, we not only confirm and visualize previously discovered common endocrine- and cell specific- enhancer regions across differentially enriched chromatin, but identify novel regions as well. We compiled our chromatin accessibility data in a freely accessible database of common endocrine- and cell specific-enhancer regions that can be navigated with minimal bioinformatics expertise.

**Conclusions:**

Both alpha and delta cells appear poised, but repressed, from becoming beta cells in murine pancreatic islets. These data broadly support earlier findings on the plasticity in identity of non-beta cells under certain circumstances. Furthermore, differential chromatin accessibility shows preferentially enriched distal-intergenic regions in beta cells, when compared to either alpha or delta cells.

**Supplementary Information:**

The online version contains supplementary material available at 10.1186/s12864-023-09293-6.

## Introduction

The evaluation of the transcriptional landscape through high-throughput bulk RNA sequencing in both mouse and human of major islet cell types has granted a deeper understanding of cellular identity and intercellular crosstalk within the pancreas. This has enabled the detection of distinct gene pattern signatures between major islet cell types in mouse and human [1–6]. However, gene expression represents the final outcome of a complex layer of genetic and epigenetic factors that determine islet cell fate [7–9] and identity [10, 11]. Previous studies have explored pancreatic islet cellular identity by evaluating epigenomic features such as methylation [12–14], histone modifications [15–18], and enhancer regulatory regions [19–24]. While each of these factors contributes to defining and maintaining cell fate and identity, connecting chromatin accessibility differences to epigenetic factors promises to provide further insight into outstanding questions within the field.

Chromatin remodeling is a central epigenetic regulator that can be surveyed in order to better understand cell states [20, 25–28]. The accessibility of chromatin via changes between euchromatin and heterochromatin, and nucleosome occupancy, plays a significant role in cell lineage determination and in tissue- and cell-specific gene expression [11, 25, 29]. Epigenetic stability is required for the maintenance of islet cell identity, while changes in chromatin accessibility are associated with perturbations in gene expression due to disease [7, 22, 30]. Chromatin accessibility in tandem with other epigenetic factors at promoter-proximal regions [29, 31] of a gene allows for direct activation or repression of transcription. In contrast, open chromatin at exonic [32], intronic [33], or distal-intergenic regions [34] can be accessed by regulatory factors that act as nearby or distal enhancers that govern lineage branching and stable cell fate.

Assay for transposase-accessible chromatin using sequencing (ATAC-Seq) allows for the unbiased, modification-independent evaluation of chromatin accessibility within cell types and can be run with relatively small sample input [30, 35]. Previous studies have explored transcriptional regulation in healthy and T2D human islet cells. Some of these studies have employed ATAC-Seq to specifically address chromatin accessibility differences in healthy [5, 11, 36, 37] and T2D [22, 23, 38, 39] human islets or human pancreatic progenitors [9] using bulk RNA-Seq through human antibody panels alongside FACS-purification or through single-cell sequencing (scATACSeq) [40–42]. However, none of these studies have explored pancreatic islet cell chromatin accessibility from mouse FACS-purified alpha, beta, and delta cells. Therefore, to better understand endocrine islet cell identity between mouse alpha, beta, and delta cells, we compared chromatin accessibility and transcriptome data for FACS-purified mouse alpha, beta, and delta cells sorted from pancreatic islets from triple transgenic reporter strains—﻿mIns1-H2b-mCherry beta cells crossed to mice with alpha or delta cells marked by YFP in a Cre-dependent fashion—that we generated for this purpose [1, 6]. This approach allowed for the direct comparison between ATAC-Seq and RNA-Seq datasets from alpha, beta, and delta cells we previously published from these exact same reporter lines [1, 6].

We integrated our ATAC-Seq data with high-quality transcription factor and histone binding data from other mouse pancreatic islet studies to evaluate how transcriptional activators and repressors may collectively regulate differential gene expression at promoter-proximal regions. To support the visualization and integration of our ATAC-Seq chromatin data and previously published transcriptome of the FACS-purified alpha, beta, and delta cells alongside select epigenomic datasets from histone marker and transcription factor Chromatin Immuno Precipitation (ChIP) data, we developed an R package, *epiRomics* [See: https://github.com/Huising-Lab/epiRomics]. This package is a novel, publicly available resource that is described in detail elsewhere [43]. In short, *epiRomics* allows for the visualization of integrated epigenomic data and visualizes putative enhancer regions without the requirement for extensive bio-informatics experience, with the intent of enabling more of our colleagues to tease apart key regions that may drive cell fate switching and maintenance between the major islet endocrine cell types. Through this approach we identified putative enhancer regions at distal-intergenic regions common to all 3 cell types as well as regions selectively accessible only in a single islet cell type and confirmed previously identified mouse pancreatic islet enhancers.

## Methods

### Experimental animals

All mouse experiments were approved by the UC Davis Institutional Animals Care and Use Committee and were performed in compliance with the Animal Welfare Act and the Institute for Laboratory Animal Research Guide to the Care and Use of Laboratory Animals. Animals were humanely euthanized using IACUC-approved methods of euthanasia in accordance with the American Veterinary Medical Association Guidelines for the Euthanasia of Animals. Mice were maintained in group housing (up to 4 mice per cage) on a 12 h light:12 h darkness cycle with water and standard rodent chow provided ad libitum. We adhered to the ARRIVE 2.0 guidelines for pre-clinical animal reporting where applicable. Our experimental design did not call for a control group as we determined the chromatin accessibility between different endocrine cells of the same tissue from the same animals. No animals were excluded from the experiments, and we observed no unexpected adverse events in the animals reported in this study. Blinding and randomization were not applicable to our study.

### Islet isolation and FACS sorting

mIns1-H2b-mCherry [1] x Rosa-LSL-YFP crossed to either Sst-Cre [44] or Gcg-Cre [45] triple transgenic mice were pooled by sex (3 male and 5 female mIns1-H2b-mCherry x Gcg-Cre x Rosa-LSL-YFP and 2 male and 5 female mIns1-H2b-mCherry x Sst-Cre x Rosa-LSL-YFP triple transgenic mice), each sample yielding a median of 20,000 cells, with islet isolation and FACS-sorting as previously described (Fig-S1) [1, 46–48].

All mouse experiments were approved by the UC Davis Institutional Animals Care and Use Committee and were performed in compliance with the Animal Welfare Act and the Institute for Laboratory Animal Research Guide to the Care and Use of Laboratory Animals. Animals were humanely euthanized using cervical dislocation as an IACUC-approved methods of euthanasia in accordance with the American Veterinary Medical Association Guidelines for the Euthanasia of Animals.

### Assay for transposase-accessible chromatin using sequencing

Single-end 50 bp reads were generated after library size selection yielded an average of 450 bp fragments and sequenced as previously described using NexteraDNA library protocol [30].

### Alignment and differential peak calling

Reads from each replicate (Table-S1) were evaluated for quality control and trimmed using FastQC and Trimmomatic, respectively [49–51]. A modified index of Gencode GRCm38.p4 (mm10) was built to exclude mitochondrial DNA prior to aligning reads with Bowtie 2 [52, 53]. Post-alignment, duplicates were marked using Picard Tools, blacklist regions were removed, and BAM files were converted into tagAlign format for downstream use. Peak calling and normalized bigwig generation was done using MACS2 [54]. Differential expression testing was performed using DiffBind’s edgeR method [55, 56].

### Quality control and validation

**Fig. 1:**
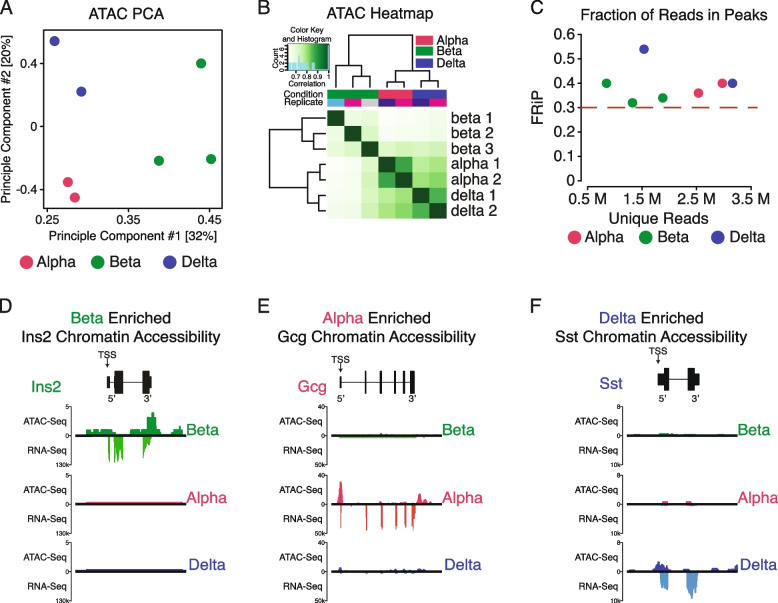
Validating alpha, beta, and delta chromatin accessibility ATAC Seq. **A** Dimensional reduction through principal component analysis across seven samples from all three cell types (See Table-S1 for details). All three cell type’s replicates clustered closer together and separate from other cell types. **B** Heatmap further confirming quality of replicates and similarity between replicates within each cell type. **C** Fraction of Reads in Peaks (FRiP) score evaluation across samples, confirming high library complexity irrespective of depth of sequencing. **D-F** Confirming chromatin normalized accessibility at the TSS (arrows) against normalized bulk RNA-Seq expression in key islet cell type-specific marker gene regions—*Ins2, Gcg,* and *Sst*—in beta, alpha, and delta cells, respectively

Quality control metrics were evaluated within raw reads as well as peak calls and compared against ENCODE standards for fraction of reads in peaks (FRiP), leading to the removal of one beta cell replicate with a FRiP score far below 0.3 (Table-S1; Fig. 1A-C) [57, 58].

### Downstream analysis

Transcription factor motif analysis and validation against existing ChIP data was performed through a modified script utilizing chromVar [59], regioneR [60], GenomicRanges [61], and motifmatchr [62] using the HOCOMOCO database [63]. Pathways analysis on differential chromatin accessibility was performed using the R Bioconductor packages ChIPseeker [64], ReactomePA [65], and clusterProfiler [66]. Gene expression and ATAC-Seq congruence testing was performed using log2FC values from both differential, companion analyses, and visualized using the R package pheatmap. Evaluation of gene expression changes across cell types was performed on RPKM values processed used Cluster 3.0, and visualized using the R package pheatmap.

### Enhancer identification

We developed a novel R package, *epiRomics*, to integrate our chromatin accessibility data alongside aggregated pancreatic islet ChIP and histone data to identify putative enhancer regions, as described [43]. The package, example data, and vignette can be found at: https://github.com/Huising-Lab/epiRomics and an interactive browser of the results from this manuscript is publicly available at: https://www.huisinglab.com/epiRomics_2021/index.html.

### Integrated data

Mouse alpha, beta, and delta (GEO: ﻿GSE80673), alongside alpha- and delta- transdifferentiated beta (GEO: GSE88778) transcriptomes were integrated into this analysis [6, 67]. Aggregated ChIP datasets of transcription factors and histone marks were added to the analysis through *epiRomics* [43] to identify putative enhancer regions (Table-S2).

## Results

### ATAC-Seq validation

To determine whether chromatin accessibility patterns differed between islet endocrine cell types, principal component analysis (PCA) was applied to peak calls across all samples. This confirmed that replicates clustered by cell type (Fig. 1A), a finding that was further validated by heatmaps using all defined peaks across replicates (Fig. 1B). Alongside quality control applied through the generation and analysis of this dataset, the fraction of reads in peaks (FRiP) score was in excess of the commonly applied benchmark of 30% (Fig. 1C). Furthermore, the FRiP score was independent of variability in unique read depth, indicating that peak calls were reproducible across all replicates within cell types independent of read depth range.

### Validation of islet cell chromatin accessibility data coupled to companion transcriptomes

**Fig. 2:**
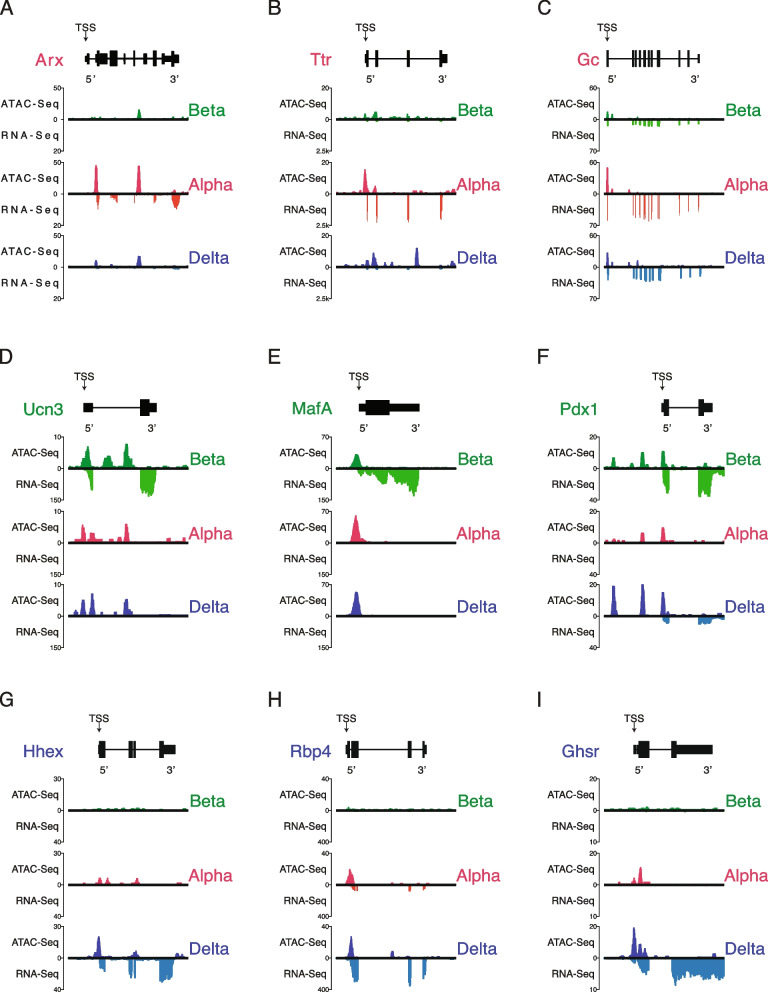
Validating chromatin accessibility ATAC Seq alongside companion RNA-Seq expression in alpha, beta, and delta cells against hallmark genes governing its respective cell’s identity. All genes are oriented for 5’ to 3’ end. **A-**C Normalized chromatin accessibility and transcript expression across alpha cell hallmark genes *Arx, Ttr,* and *Gc*. **D-F** Normalized chromatin accessibility and transcript expression across beta cell hallmark genes *Ucn3, Esr1,* and *Pdx1.* G-I: Normalized chromatin accessibility and transcript expression across delta cell hallmark genes *Hhex, Rbp4,* and *Ghsr*

We then checked for the presence of chromatin peak enrichment for established alpha, beta, and delta marker genes. We expected enriched ATAC signal at promoter-proximal regions near the transcription start site (TSS) as a reflection of chromatin accessibility. Indeed, cell type-specific chromatin accessibility correlated with gene expression of *Ins2*, *Gcg*, and *Sst* genes for beta, alpha, and delta cells, respectively (Fig. 1D-F) [4, 6, 68]. After confirming chromatin accessibility in key cell-identity markers, we sought to compare and contrast select regions identified from prior groups that evaluated chromatin accessibility in human islets [5, 12, 38], as well as to further query whether chromatin was always uniquely enriched on a panel of cell type-specific genes across alpha (*Arx, Ttr, Gc)*, beta (*Ucn3, MafA, Pdx1)*, and delta cells (*Pdx1, Hhex, Rbp4, Ghsr*) (Fig. 2; Fig-S2). Many of these genes demonstrated overall strong concordance between cell type-enriched gene expression and cell type-specific enrichment of available chromatin. For some of these genes – notably MafA, Pdx1, and Gc, the chromatin at the TSS is accessible in multiple islet cell types, while the corresponding transcripts are more restricted. This validated the utility of ATAC-Seq data to detect determinants of gene expression.

### The chromatin landscape of the annotated genome across cell types

**Fig. 3:**
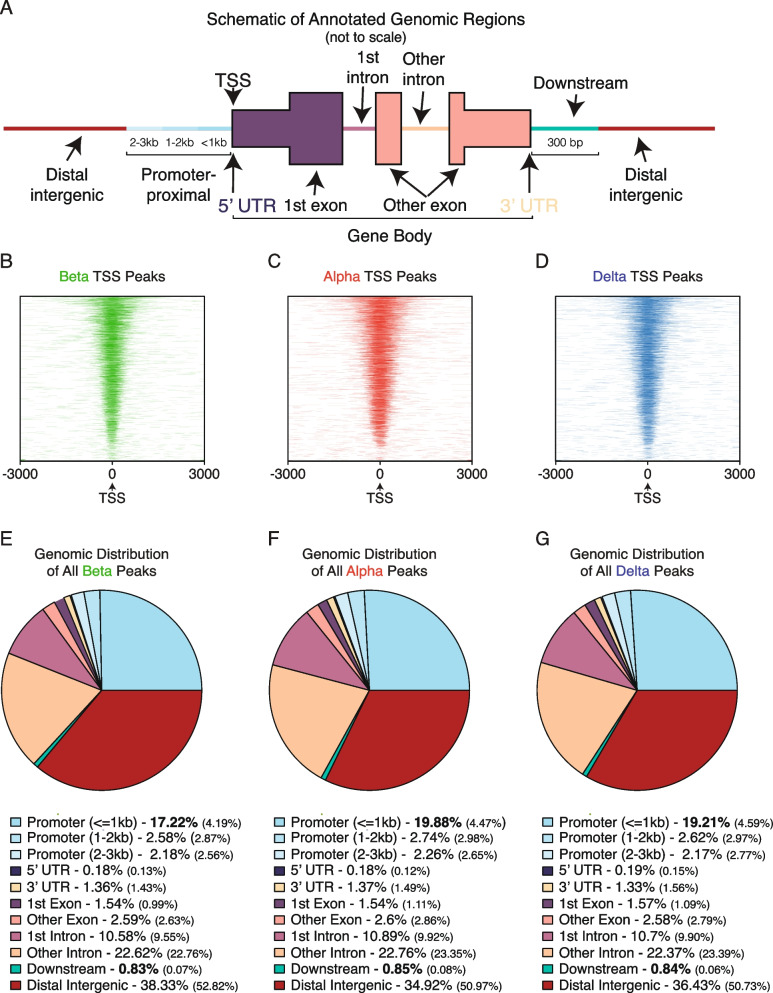
Evaluating chromatin accessibility ATAC Seq similarities and differences across all three cell types. **A** Schematic of annotated genomic regions – promoter proximal, intronic, exonic, distal-intergenic, or downstream. **B-D** TSS peak (defined as 3 kb up or downstream each respective gene) chromatin accessibility density across beta, alpha, and delta cells. **E–G** Distribution of chromatin peaks within each cell type across the annotated genome against a background genome distribution (in parenthesis)

As genes make up a small fraction of the entire genome, we determined the overall distribution of peaks across the annotated genome within each cell type. We defined five regions of interest to further explore – promoter-proximal, intronic, exonic, downstream, or distal-intergenic (Fig. 3A). We identified a consensus set of 124,494 peaks marking open chromatin through the R package DiffBind. This number is comparable to the number of open regions found in previous studies of pancreatic islet chromatin accessibility [11, 38–40] (Dataset-S1). We then evaluated the distribution of called peaks present in at least two replicate within 3 kb upstream of the TSS and confirmed that a majority of genes enriched in each islet cell type were accompanied by promoter-proximal peaks (Fig. 3B-D). The distribution of ATAC-Seq peaks across different pre-defined genomic areas was overall similar across alpha, beta, and delta cells. For each endocrine cell type between 21.98–24.88% of open chromatin was promoter-proximal, whereas promoter-proximal areas account for 2.41% of the mouse genome. A further 34.92–38.33% of peaks for all cell types were found on distal-intergenic regions (Fig. 3E-G). Finally, we noted that between 33.07- 33.65% of peaks occurred on intronic regions (first or other), relative to the 37.7% of the mouse genome classified as intronic [69].

### Regional differences and characteristics of differentially enriched chromatin

**Fig. 4:**
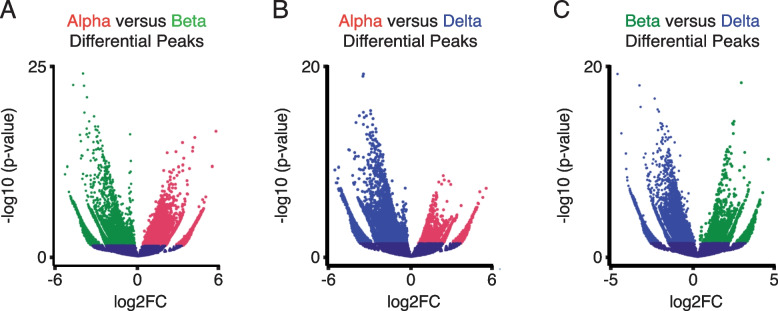
Comparing chromatin accessibility through differential enrichment analysis across alpha, beta, and delta cells. **A** Differential chromatin accessibility peaks between alpha and beta ATAC Seq data. A total of 18,409 peaks were considered differentially enriched at *p*-value <  = 0.05 (Dataset-S1). **B** Differential chromatin accessibility peaks between alpha and delta ATAC Seq data. A total of 12,722 peaks were considered differentially enriched at *p*-value <  = 0.05 (Dataset-S1). **C** Differential chromatin accessibility peaks between beta and delta ATAC Seq data. A total of 16,913 were considered differentially enriched at *p*-value <  = 0.05 (Dataset-S1)

As our overall distribution of ATAC-Seq peaks across different genomic regions was consistent across alpha, beta, and delta cells, we compared differential chromatin accessibility between these cell types in greater detail. To this end, we performed pairwise differential ATAC-Seq peak enrichment testing across alpha, beta, and delta cells. Out of 124,494 identified consensus regions of open chromatin across the three cell types, 18,409 (14.8%) differentially enriched peaks (*p*-value <  = 0.05) were identified between alpha and beta (Fig. 4A), 12,722 (10.2%) between alpha and delta (Fig. 4B), and 16,913 (14.6%) between beta and delta cells (Fig. 4C).

**Fig. 5:**
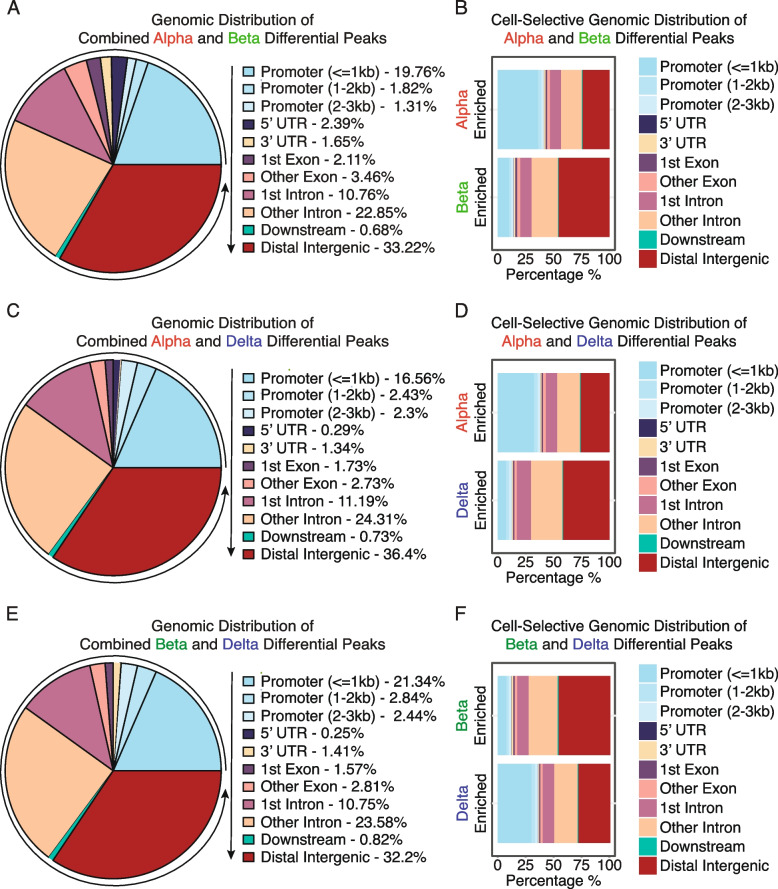
Regional differences and characteristics of differentially enriched peaks between alpha, beta, and delta cells. **A** Distribution of regional preference across the annotated genome of differentially enriched peaks between alpha and beta cells. **B** Regional preference breakdown of differentially enriched peaks between alpha and beta cells, indicating prevalence of enrichment for each cell type and genomic annotation. Differentially enriched chromatin favored promoter-proximal peaks in alpha cells, and distal-intergenic regions in beta cells. **C** Distribution of regional preference across the annotated genome of differentially enriched peaks between alpha and delta cells. **D** Regional preference breakdown of differentially enriched peaks between alpha and delta cells, indicating prevalence of enrichment for each cell type and genomic annotation. Differentially enriched chromatin favored promoter-proximal peaks in alpha cells, and distal-intergenic regions in delta cells. **E** Distribution of regional preference across the annotated genome of differentially enriched peaks between beta and delta cells. **F** Regional preference breakdown of differentially enriched peaks between beta and delta cells, indicating prevalence of enrichment for each cell type and genomic annotation. Differentially enriched chromatin favored promoter-proximal peaks in delta cells, and distal-intergenic regions in beta cells

After performing differential peak enrichment testing, we discovered that 22.89% of all differentially enriched peaks between alpha and beta cells were promoter-proximal (0-3 kb) (Fig. 5A). A further 33.22% of differential peaks were linked to distal-intergenic regions and another 33.61% of differential peaks were intronic (first and other combined) (Fig. 5A). This assessment of differential peaks without considering the direction of enrichment revealed no major difference with overall peak distribution described earlier (Fig. 3). However, when factoring in the direction of enrichment we observed that 35.08% of alpha cell-enriched peaks was promoter-proximal. In contrast, only 12.5% of beta cell enriched peaks occurred in promoter-proximal areas (Fig. 5B). Instead, a majority of ATAC peaks enriched in beta cells were located at distal-intergenic regions (45.41%) (Fig. 5B).

Between alpha and delta cells, we identified that 21.29% of differentially enriched peaks occurred promoter-proximally. Another 36.4% of peaks occurred on distal intergenic regions and 35.5% on intronic regions (Fig. 5C). A similar preference of alpha cell-enriched peaks in promotor-proximal regions was evident when comparing alpha to delta cells, with 30.33% of all enriched alpha peaks occurring promoter-proximally, but only 9.56% of delta cell peaks. Instead, 38.41% of delta cell enriched peaks were distal-intergenic (Fig. 5D).

Lastly, between beta and delta cells, 26.62% of all differentially enriched peaks were promoter-proximal, 32.2% distal intergenic, and 34.33% on intronic regions (Fig. 5E). Further break down revealed a bias towards distal-intergenic enriched peaks within beta cells (42.86%), as opposed to promoter-proximal peaks in delta cells (28.20%) (Fig. 5F).

### Differential chromatin enrichment in the majority of cases correlates with gene expression

**Fig. 6:**
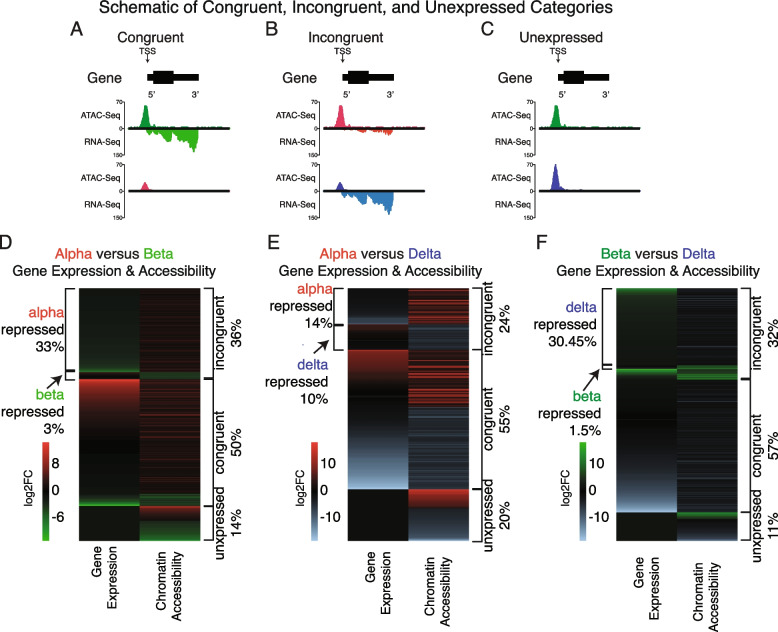
Differentially enriched chromatin at TSS genic regions and their respective gene’s expression between alpha, beta, and delta cells. **A-C** Schematic of ‘congruent’, ‘incongruent’, and ‘unexpressed’ categories used to determine the association of enriched chromatin at TSS genic regions and respective gene expression. **D** Differentially enriched chromatin at TSS genic regions and their respective gene’s expression between alpha and beta cells. The majority (50%) of genes with enriched chromatin at promoter-proximal regions around their TSS had correlated gene expression (congruent). Another 36% of chromatin enriched TSS regions showed repressed gene expression for each cell type (alpha repressed (33%) or beta repressed (3%)), and finally, 14% were unexpressed. **E** Differentially enriched chromatin at TSS genic regions and their respective gene’s expression between alpha and delta cells. The majority (55%) of genes with enriched chromatin had correlated gene expression (congruent). Another 24% of chromatin enriched TSS genic regions showed repressed gene expression for each cell type (alpha repressed (14%) or delta repressed (10%)), and finally, 20% showed no expression. **F** Differentially enriched chromatin at TSS genic regions and their respective gene’s expression between beta and delta cells. The majority (57%) of genes with enriched chromatin had correlated gene expression (congruent). Another 32% of chromatin enriched TSS regions showed repressed gene expression for each cell type (beta repressed (1.5%) or delta repressed (30.45%)), and finally, 11% showed no difference

So far, we detected a disproportionate fraction of peaks associated with promoter-proximal regions in general, compared to the fraction of the genome that consists of this type of region (Fig. 3). Moreover, ATAC-Seq peaks that were differentially enriched in alpha and—to a lesser extent—delta cells were considerably more likely to occur at promoter-proximal sites. Instead, peaks enriched in beta cells more likely occurred at distal intergenic regions (Fig. 5). Therefore, we determined whether the enrichment of promoter-proximal peaks correlated with increased expression of the corresponding genes. Genes with increased expression in a cell type accompanied by a significantly enriched ATAC-Seq peak proximal to its TSS were considered ‘congruent’ genes (Fig. 6A). The underlying mechanism in such a scenario might be the presence of transcriptional activators at the promoter-proximal site that promote gene expression. Conversely, genes with a significantly enriched ATAC-Seq peak proximal to its TSS accompanying a reduction in corresponding gene expression were considered ‘incongruent’ genes (Fig. 6B). The underlying mechanism for these genes might be the presence of transcriptional repressors at the promoter that prevent gene expression (Dataset-S2) [70–73]. Finally, genes that had significantly enriched chromatin in either cell type, but no evidence of mRNA expression were considered ‘unexpressed’ (Fig. 6C).

When we compared differentially enriched TSS-associated chromatin against corresponding gene expression between alpha and beta cells, we found that in the majority of cases (86%), differential chromatin enrichment on TSS regions positively correlated with changes in gene regulation. Exactly, 50% of genes with differentially enriched chromatin at the TSS had a corresponding increase in gene expression within the same cell type (congruent genes; Fig. 6D). 36% showed TSS chromatin accessibility enrichment, but with a reduction in gene expression for each cell (incongruent genes—either alpha repressed (33%), or beta repressed (3%)). Strikingly, a substantial majority of the incongruent genes in this comparison were alpha repressed. Finally, only 14% of all genes with differentially enriched TSS chromatin showed no expression in either cell type (unexpressed) (Fig. 6D).

We highlight the beta-specific gene *MafA* as an example of an incongruent gene that is putatively alpha repressed or alpha cell poised (Fig. 2E; Fig-S3A)*.*

We observed a similar distribution between congruent (55%), incongruent (24%), and no expression genes (20%), between alpha and delta cells. We noted a more uniform distribution between alpha (14%) and delta (10%) repressed genes. (Fig. 6E). Upon visualizing gene expression and chromatin accessibility, we confirmed congruent gene expression and TSS chromatin accessibility of key transcription factors known to regulate both alpha – *MafB*, *Ttr*, and *Arx* – and delta – *Pdx1* and *Hhex* – cell fate (Fig-S3B).

For the pairwise comparison between beta and delta cells, we again found a similar fraction of congruent (57%), incongruent (32%), and no expression (11%) genes (Fig. 6F). We noted a minor fraction of repressed genes with open chromatin in beta cells (1.5%), with the majority of repressed genes corresponding to delta cells (30.45%), similar to the pattern seen in alpha repressed genes between alpha and beta cells. Further visualization of select marker gene expression against chromatin accessibility showed generally good congruence between chromatin accessibility at the TSS and gene expression (Fig-S3C)*.*

### Poised genes are enriched in beta cells with a non-beta cell lineage history

**Fig. 7:**
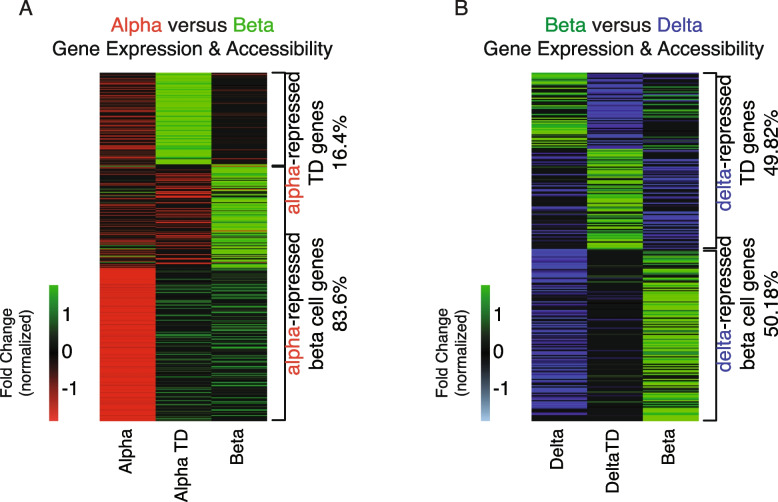
Gene expression of poised genes enriched in beta cells with a non-beta cell lineage. **A** Evaluating alpha repressed genes (Fig. 6A) across alpha, alpha transdifferentiated, and beta cell transcriptomes. The great majority (83.6%) of genes repressed in alpha cells showed intermediate expression in alpha transdifferentiated cells, and highest expression in beta cells, further validating that alpha cells are poised to become beta cells, with a subset (16.4%) of those genes required for the transition. **B** Evaluating delta repressed genes (Fig. 6C) across delta, delta transdifferentiated, and beta cell transcriptomes. Around half (50.18%) of genes repressed in delta cells showed intermediate expression in delta transdifferentiated cells, and highest expression in beta cells, validating that delta cells – to a lesser extent than alpha – are also poised to become beta cells, with the remainder of genes (49.82%) required for the transition

Since most of the incongruent genes are assigned to alpha and delta cells in their respective comparisons with beta cells, we further interrogated whether these alpha- or delta- repressed genes could be poised beta cell genes. In order to do so, we incorporated transcriptome data from beta cells with an alpha- or delta- cell lineage history [67] – also from our companion RNA-Seq experiment. Unfortunately, there is no accompanying ATAC-Seq data for these cells. These cells, termed “transdifferentiated,” are beta cells (defined by the presence of *Ins1-driven mCherry expression*), but have either a *Gcg-* or *Sst*-Cre lineage label, reflective of a lineage history as an alpha or delta cell, respectively. We reasoned that if alpha- or delta- repressed genes are poised beta cell genes, we should expect to observe a stepwise transition in gene expression levels, showing little or no expression in either alpha or delta cells, to intermediate expression in alpha- or delta- transdifferentiated cells, and full expression in beta cells. We confirmed that the majority (83.6%) of alpha-repressed genes showed intermediate expression in the alpha-to-beta-transdifferentiated population, and the highest expression in beta cells (beta cell genes). A subset of genes (16.4%) showed the highest expression in the alpha-transdifferentiated population (Fig. 7A). We observed a similar pattern between delta, delta-transdifferentiated, and beta cells; however, only half (50.18%) of delta repressed genes demonstrated an intermediate expression in the delta-to-beta-transdifferentiated population and the highest in beta (Fig. 7B). The remainder of the genes showed the highest expression in delta-to-beta-transdifferentiated cells.

### Differential meta-chromatin enrichment testing

Given that for a majority of genes, TSS-associated chromatin recapitulated the underlying regulation of gene expression, we inquired whether differentially enriched chromatin peaks were associated with genes concentrated in pathways or gene networks that would better reflect our understanding of the biology across these different islet endocrine cell types. Between alpha and beta cells, KEGG set pathway testing of differentially accessible chromatin identified pathways related to protein digestion and absorption and cell adhesion molecules unique to beta cells; Hippo, Wnt, and ubiquitin-mediated proteolysis unique to alpha cells; and MAPK, axon guidance, and cAMP pathways enriched within both (Fig-S4A-B). Upon comparing the differentially accessible chromatin between alpha and delta cells, adherens junctions were enriched in delta cells, while no pathways were enriched specifically in alpha cells. MAPK, axon guidance, and Ras signaling pathways showed general enrichment of associated peaks within both alpha and delta cells (Fig-S5A-B). Lastly, in beta and delta cells, a pairwise analysis of differentially accessible chromatin identified the Glycosaminoglycan (GAG) biosynthesis pathway as unique to beta cells—where GAG metabolism and biosynthesis impairment has been linked to beta cell dysregulation [74], adherens junctions and Rap1 signaling pathways unique to delta cells, and MAPK, axon guidance, and cAMP signaling pathways enriched within both, as well as identified between alpha and beta cells (Fig-S6A-B).

### Islet transcription factor ChIP-Seq binding correlates with open chromatin

After exploring the interrelationship between accessible chromatin and gene expression, we expanded our approach to include additional controls to the regulation of islet cell gene expression. We therefore aggregated high-quality, mouse pancreatic islet transcription factor binding data via ChIP-Seq—Pdx1 [75], Nkx6-1 [76], Neurod1 [77], Insm1 [77], Foxa2 [77], Nkx2-2 [78], Rfx6 [79], MafA [24], Isl1 [80], Kat2b [81], Ldb1 [80], and Gata6 [82]—and asked what fraction of open chromatin – as defined by our consensus ATAC-Seq peak set – contains binding sites for each respective transcription factor. Of all open chromatin sites, individual transcription factors occupied a significant percent with Foxa2 (29.07%), Insm1 (28.40%), and Neurod1 (20.09%) showing the highest percentage of ChIP-confirmed binding. This provided further support that our ATAC-Seq data was of high quality and suggested that open chromatin is a reliable indicator of epigenetic regulation (Table-S3A). To further explore whether these aggregated transcription factor ChIP data correlated with open chromatin, we queried what fraction of total ChIP binding sites overlapped with ATAC-Seq peaks. Indeed, we observed that in several cases, over 50% of transcription factor ChIP binding sites overlapped with open chromatin, with the transcription factors Nkx2.2 (63.79%), Neurod1 (55.07%), and Insm1 (51.49%) showing the greatest degree of overlap (Table-S3B). These results supported our findings that open chromatin indicates regulation of gene expression in alpha, beta, and delta cells, in part through the binding of transcription factors.

### Transcription factor motif finding suggests genomic preferences at differentially enriched chromatin between cell types

After observing a strong degree of overlap of known islet transcription factor binding on open chromatin, we conducted an unbiased evaluation whether DNA motifs for their respective transcription factor proteins were differentially enriched on ATAC peaks in promoter, intronic, exonic, downstream, or distal regions. We included only transcription factors with known DNA-binding motifs to determine if they were more likely to occur at specific areas of the genome. We required that the transcription factor associated with the DNA sequence motif considered is expressed (RPKM > 0) in the cell type with chromatin-motif association.

**Fig. 8:**
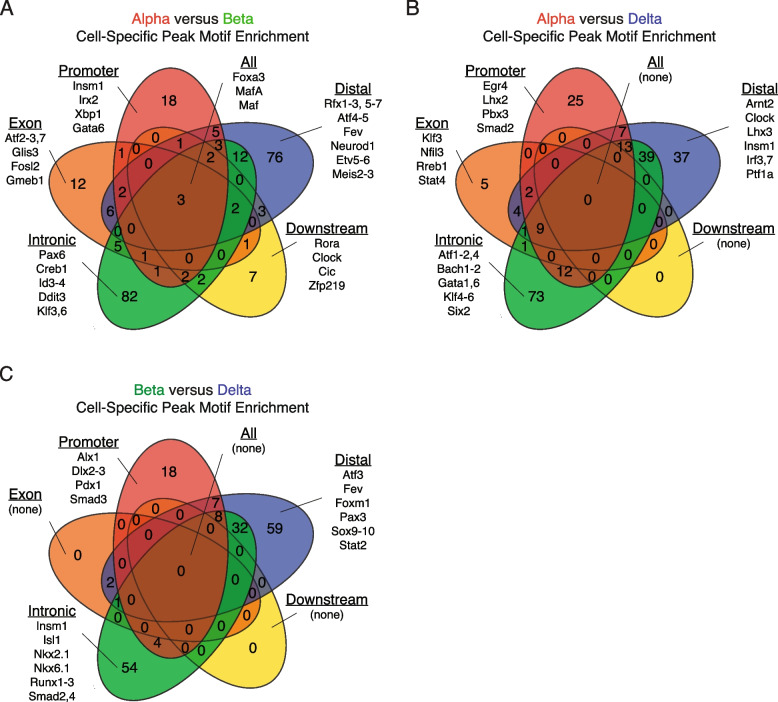
Evaluating expressed, cell-specific transcription factor motifs on differentially enriched peaks across cell types. **A** Evaluating cell-specific transcription factor motifs associated with differentially enriched peaks for alpha and beta cells, suggesting transcription factor preference for these peaks across the functionally annotated genome. Notably, three known transcription factors were predicted to overlap all defined regions of the genome, whereas others showed preference for binding at either promoter, exon, intron, or distal regions, suggesting different mechanisms of regulation. **B** Evaluating cell-specific transcription factor motifs associated with differentially enriched peaks for alpha and delta cells, suggesting transcription factor preference for these peaks across the functionally annotated genome. No known transcription factor was predicted to bind to all defined regions of the genome, with the majority binding to either intronic, distal, or promoter areas. **C** Evaluating cell-specific transcription factor motifs associated with differentially enriched peaks for beta and delta cells, suggesting transcription factor preference for these peaks across the functionally annotated genome. No known transcription factor was found predicted to bind to all defined regions of the genome, with the great majority showing a preference for distal, intronic, or promoter regions

Motifs for key transcription factors involved in beta cell identity, such as MafA, were present ubiquitously across most functional regions we defined (promoter-proximal, intronic, exonic, downstream, and distal intergenic) (Fig. 8A). In contrast, the motifs for alpha cell-identity driver Irx2 [4] were concentrated at the promoter-proximal regions of chromatin peaks associated with genes differentially expressed by alpha cells. Insm1 [77] motifs were concentrated at the promoter-proximal regions of chromatin peaks associated with genes differentially expressed by beta cells. In another example, DNA-binding motifs associated with the ubiquitous islet transcription factor Pax6 [83] were concentrated on intronic chromatin.

We performed the same transcription factor motif enrichment test between alpha and delta cells (Fig. 8B). Of note, the motif for Pbx3, a transcription factor driving *Sst* expression in delta cells [84], was enriched in accessible chromatin at promoter-proximal regions. The motif for Stat4, recently implicated in establishing alpha cell identity [85] was concentrated in exonic chromatin. Lastly, the motif for Ptf1a, a transcription factor identified in early pancreatic endocrine cell development [86], was preferentially associated with areas of open chromatin at distal intergenic regions.

Between beta and delta cells, no single transcription factor motif overlapped across all five functional categories, nor were there any unique to downstream or exonic regions, as we observed in the prior alpha and delta comparisons (Fig. 8C). Of note, the motif for Smad3*,* a transcription factor important for islet development [87], as well as the negative regulation of insulin secretion in beta cells via occupancy of the insulin promoter [88], was concentrated in promoter-proximal accessible chromatin*.* Motifs for Insm1 [77] and Nkx6.1 [89], both key beta cell identity transcription factors, were preferentially associated with accessible chromatin at intronic regions. Lastly, motifs for Fev – recently identified as important for the development and differentiation of the endocrine lineage [90]—and Atf3* –* linked to enhancer regions in ﻿EndoC-bH1 cells [11]—were enriched in accessible chromatin at distal regions.

### Validating motif calls against aggregated islet ChIP datasets

As we observed motif binding site preferences across promoter, intronic, exonic, downstream, or distal chromatin regions, we wished to confirm how accurately predictive DNA motif binding sites conveys true transcription factor binding. To do so, we once again turned to our aggregated pancreatic islet ChIP datasets. We applied the same motif detection method as above on individual ChIP datasets and on all open chromatin – as derived from our ATAC-Seq consensus peak set –and assessed how well predicted motif binding overlapped with true ChIP peaks from our selected list of ChIP-Seq data. One transcription factor, Rfx6, showed strong (57%) true positive and low (8.34%) false negative values for its DNA-motif’s ability to predict all Rfx ChIP binding sites (Table-S4A). We then limited this same test to Rfx6 ChIP-Seq binding sites (35.65%) that were shared across 1.19% of all open chromatin ATAC peaks (Table-S3A-B). Notably, when comparing DNA-motif predictions for Rfx6 against these shared Rfx6 ChIP-Seq binding sites, we observed that 65.10% were true positives, while 8.81% were false predictions (Table-S4B). When we expanded this analysis to other transcription factors, we observed a broad distribution in true positive values across these DNA-binding motifs (4.71–65.10%), and also noted a relatively low range of false positives (1.68–8.81%). This is reflective of a limitation in the ability for DNA-motifs to consistently predict true transcription factor binding.

### Determining overlap of differentially enriched chromatin with islet ChIP and histone datasets

Given that many of the DNA-motifs associated with transcription factors do not have available ChIP-Seq datasets derived from mouse pancreatic islets, we sought to understand whether differential chromatin between cell types could be associated to transcription factors and histone markers integral to pancreatic islet cell fate that do have available ChIP data, as opposed to relying only on predictive motifs. As we previously observed strong enrichment of transcription factor binding sites across all open islet chromatin, we wanted to confirm if this overlap is augmented in differentially enriched chromatin associated with pancreatic islet transcription factor binding sites via aggregated islet ChIP data—Pdx1, Nkx6-1, Neurod1, Insm1, Foxa2, Nkx2-2, Rfx6, and MafA—and select, key histone marks — H3K27ac [91], H3K4me3 [91], and H3K4me1 [12]. Our intention in integrating these data was in anticipation that they may help delineate whether enhancer regions are poised (defined by: H3k4me1) [92], or active (defined by: H3k27ac and H3k4me1) [93], and whether promoter regions are active (defined by: H3k4me3) [94].Indeed, we observed that differentially enriched ATAC-Seq peaks within our comparisons occurred at much higher rates than random chance across a majority of transcription factor ChIP data associated with islet cell identity (Fig-S7A-C) as well as all predictive histone marker regions (Fig-S7D-F). This further supported our hypothesis that open chromatin, and now specifically differentially enriched chromatin, would be directly associated with the transcription factors responsible for shaping islet cell-specific gene expression patterns and identity. As differentially enriched chromatin is associated with cell-identity regulatory networks, we inquired to selectively evaluate these regions for enhancers that may be relevant to pancreatic islet cell identity.

### Identifying and visualizing putative islet cell-type specific enhancers via epiRomics

**Fig. 9:**
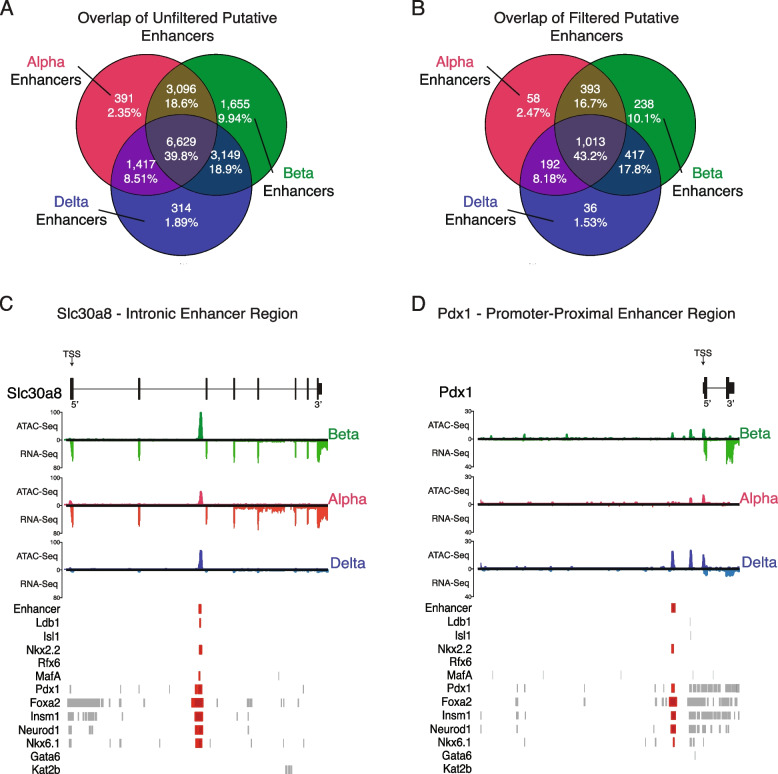
Putative enhancer detection overlap between the three cell types. **A** First-pass overlap of unfiltered putative enhancers called with our novel package, epiRomics. Open chromatin regions in at least one cell type were crossed against two informative histone marks—H3k27ac and H3k4me1 – and transcription factor binding data to call putative enhancer regions. A total of 28,647 regions were identified (Dataset-S3). 39.8% of putative enhancer calls had chromatin accessible to all three cell types, suggestive of pancreatic endocrine cell development and maintenance involvement. The overlap of enhancer calls with open chromatin between any two cells type was 8.51%—18.9%. Between 1.89%—9.94% of calls were unique to one cell type alone. **B** First-pass enhancer calls were filtered against the curated FANTOM5 database delineating all identified enhancers in the mouse genome. This resulted in a much more conservative list of 3,535 regions identified (Dataset-S4). The distribution of enhancers unique or common between cell types remained comparable, with 43.2% identified across all three cell types, and 1.53%—10.1% unique to a cell type. **C** Confirming an enhancer on the second intron of *Slc30a8*, identified in a previous study, with 14 sites of co-binding from multiple transcription factors. **D** Confirming an a promoter-proximal enhancer (~ 1 kb upstream) of the gene that codes for the transcription actor *Pdx1*, with 9 sites of co-binding from multiple transcription factors

These transcription factor and histone ChIP datasets were then fed into an R package named *epiRomics* that we developed to identify putative enhancer regions involved in pancreatic islet cell identity. We defined enhancer regions by the co-localization of H3k27ac and H3k4me1 (activating) histone modifications within islet cell chromatin. We narrowed our definition further by requiring these regions to also have transcription factor binding sites—Pdx1, Nkx6-1, Neurod1, Insm1, Foxa2, Nkx2-2, Rfx6, MafA, Isl1, Kat2b, Ldb1, or Gata6 – defined by islet ChIP data that are either ubiquitously or selectively expressed across the three islet cell types (Fig. 2E-F; Fig-S2E; Fig-S8A-I). This first pass resulted in 28,645 putative enhancer regions (Dataset-S3). We then filtered this list against chromatin accessible regions from our ATAC-Seq data sets of alpha, beta, and delta cells, resulting in 16,651 putative active enhancers (Fig. 9A). To further increase our confidence in these enhancer calls, we crossed our putative enhancer regions against the FANTOM5 curated enhancer database [95]. This resulted in a conservative list of 3,535 putative enhancer regions. Of these, 3,203 were inaccessible to at least two out of three islet endocrine cell types (Fig. 9B) (Dataset-S4).

In both putative enhancer lists, we found that 39.8–43.2% of the enhancer regions we identified were common across all cell types, supporting the theory that related cell types of a common origin would have a sizeable commonality of similar regulatory regions involved in development and maintenance (Fig. 9A-B). Interestingly, between 1.53–10.1% of called enhancers were associated with accessible chromatin unique to each cell type. Enhancer regions selective to beta cells were identified at the highest frequency (~ 10%), while alpha and delta enhancers made up ~ 2% of the list.

As a reality check, we cross referenced our putative enhancer list with several established enhancer sites that were experimentally confirmed. Our approach correctly predicted an established intronic enhancer on the *Slc30a8* gene, which was demonstrated to be regulated in part by the Pdx1 transcription factor (Fig. 9C) [96]. Our approach also correctly predicted a previously identified promoter-proximal enhancer region targeting *Pdx1*, with co-occurring binding sites for islet transcription factors Insm1*,* Neurod1*,* and Foxa2 (Fig. 9D) [77]*.* This validated the use of the epiRomics approach to predict novel, not previously identified enhancer regions.

**Fig. 10:**
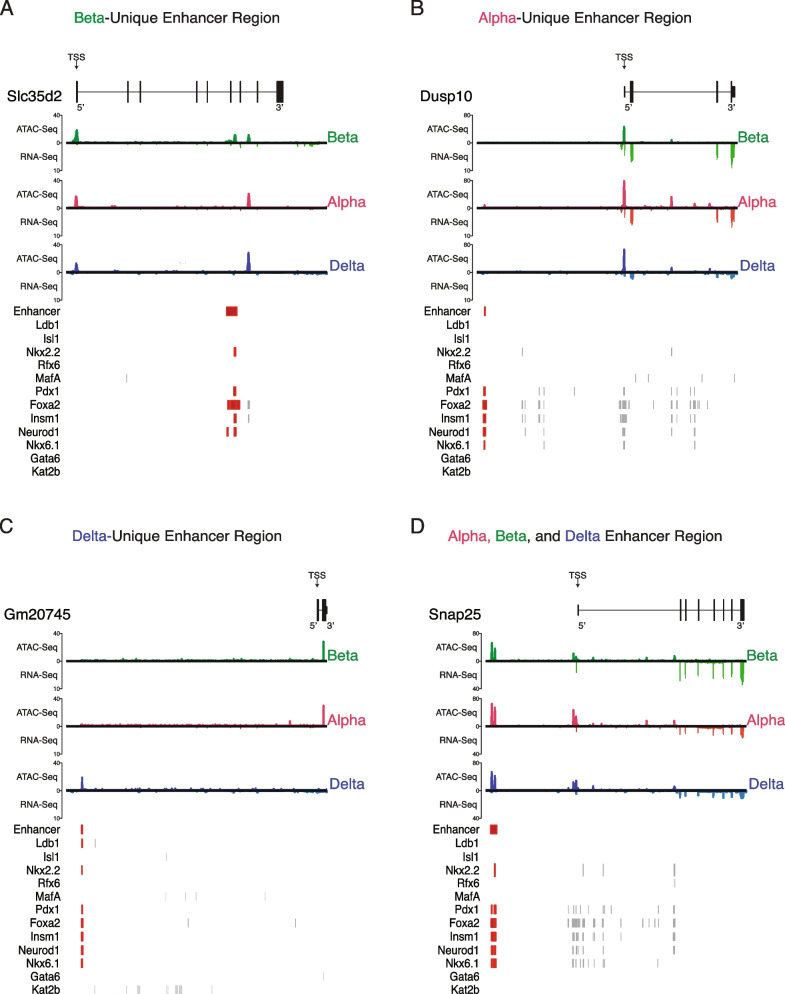
Visualizing novel, putative enhancer detection between cell types. **A** Visualizing a beta-unique enhancer region. An exonic enhancer region selected from our filtered enhancer call list, with 8 sites of co-binding from various transcription factors relevant to pancreatic islet cell identity and maintenance [1]. **B** Visualizing an alpha-unique enhancer region; a distal-intergenic enhancer region (~ 30 kb upstream of *Dusp10*) selected from our filtered enhancer call list, with 6 sites of co-binding from various transcription factors. **C** Visualizing a delta-unique enhancer region. A distal-intergenic enhancer region (~ 21 kb upstream of *Gm20745*) selected from our filtered enhancer call list, with 12 sites of co-binding from various transcription factors. **D** Visualizing a non-unique enhancer region common across all three cell types. A distal-intergenic enhancer region (~ 32 kb upstream of *Snap25*) selected from our filtered enhancer call list, with 17 sites of co-binding from various transcription factors

We investigated cell-specific or common putative enhancer candidates, starting with those with the highest number of transcription factor co-binding sites from our list. One of the top predicted beta cell-unique putative enhancer regions is located on the sixth exon of the *Slc35d2* gene and aligns with eight different ChIP co-localization binding sites (Fig. 10A). An alpha cell-unique putative enhancer located at a distal-intergenic region ~ 30 kb upstream of *Dusp10*, overlapped precisely with six sites of co-binding from various transcription factors (Fig. 10B). A delta cell-unique region at a distal-intergenic enhancer region ~ 21 kb upstream of *Gm20745* aligned closely with no fewer than 12 sites of co-binding from multiple transcription factors (Fig. 10C). And finally, a common enhancer located ~ 32 kb upstream of *Snap25* – a gene expressed in alpha, beta, and delta cells, associated with a total of 17 co-binding sites of aggregated transcription factors (Fig. 10D).

We noted further examples of enhancer regions that are inaccessible to beta, but present in both alpha and delta (Fig-S9A) cells, or others with chromatin accessibility across all cell types with an adjacent, intronic enhancer region uniquely available to beta cells alone (Fig-S9B). In particular, the *Slc2a2* gene shares common open chromatin across alpha, beta, and delta cells. However, beta cells have a gained accessible chromatin region on the first intron identified as a putative enhancer and which overlaps with six co-binding sites. Finally, we noted more putative regions that were enriched in both alpha and beta cells, and present in delta cells as well (Fig-S9C-D).

## Discussion

The high quality ATAC-Seq data derived from this study is the first dataset of its kind from FACS-purified mouse alpha, beta, and delta cells. Moreover, while bulk-ATAC-Seq data from human alpha and beta cells have previously been reported, our data are the first to report on chromatin accessibility of delta cells and combine these cell’s chromatin landscapes to companion transcriptome data. We believe that our data will provide a useful resource that complements our companion transcriptome data that we reported previously using the exact same combination of reporter strains [6]. Leveraging these data allowed us to confirm previous findings in a human ATAC-Seq study evaluating alpha and beta cells, which suggested that alpha cells are poised but repressed from becoming beta cells [5], and present evidence that supports that delta cells might be similarly poised to adopt a beta cell like gene expression pattern. We also now harmonized our ATAC-Seq and RNA-Seq data with a wealth of -omics levels data from our colleagues, resulting in a comprehensive multi-layered omics overview that includes histone modifications and transcription factor binding sites. Finally, we made these data accessible through an intuitive interface that we developed to be navigated without any bioinformatics experience (https://www.huisinglab.com/epiRomics_2021/index.html).

In evaluating the chromatin landscape of alpha, beta, and delta cells, we noted that over half of accessible chromatin in any of the cell types corresponded to promoter-proximal regions (~ 25%) and intronic regions (~ 32%), even though a much smaller fraction of the genome is represented by promoter-proximal sites. This underscores that a substantial portion of regulatory activity occurs directly at genic regions themselves. The enrichment of promoter-proximal and intronic open chromatin we observed in mouse islet cells agrees with previous findings in human studies [5, 38]. The strong presence of intronic open chromatin supported previously established findings of how enhancers on introns can act as suppressors [33] or drivers of gene expression [97]. As an example, *Pdx1* regulates the expression *Slc30a8* through an intronic enhancer [96] – which suggested that these intron regions of accessible chromatin may play a role in cell identity (Fig. 9D). Our findings of a large number of peaks residing at distal-intergenic regions (~ 35%) agree with previous research identifying and emphasizing the role of distal intergenic regions acting as enhancers (Fig. 10C-D, Fig-S9C-D) in pancreatic islet identity and functional beta cell behavior, and through linkage with T2D GWAS studies that link these regions to beta cell dysregulation [19, 20, 23, 24, 98, 99].

Upon evaluating differences in chromatin accessibility in pairwise comparisons, we discovered that overall, differentially enriched ATAC-Seq peaks in alpha or delta cells were more likely to occur at promoter-proximal regions adjacent to the TSS, whereas peaks enriched in beta cells were often found in distal intergenic or intronic regions, suggesting different mechanisms regulating alpha and delta cell fate specification (Fig. 5B, F). When comparing differentially enriched TSS-associated chromatin and respective gene expression, we observed a strong association between chromatin accessibility and gene expression. However, both alpha and delta cells showed an abundance in putatively poised genes when either was compared to beta cells. Of note, *MafA* is a key transcription factor enriched in beta cells that shows abundant chromatin accessibility in both alpha and delta cells (Fig. 2E) but is only expressed in beta cells. Another notable example is *Pdx1*, which shows repressive activity (poised TSS enrichment) in alpha cells, but is only expressed in beta and delta cells (Fig. 2F).

Moreover, a majority of alpha- and delta- repressed genes showed intermediate expression in beta cells from alpha or delta origin, respectively (Fig. 7A-B), further supporting that these are indeed putatively poised. These observations are in line with prior data that suggest that alpha cells are epigenetically poised to become beta cells, but are prevented from assuming beta cell transcriptional programs by repressive regulators at key beta-specific transcription factors [5, 15]. Our observations here also fit reports of adult or juvenile transdifferentiation of alpha-to-beta cells, or delta-to-beta cells, respectively [5, 67, 100], although a meaningful contribution of these processes to beta cell regeneration in the absence of transgenic interventions is uncertain [101, 102].

After we evaluated motif binding on differentially enriched chromatin, we found that Irx2 and Insm1 motifs are enriched at promoter-proximal regions of cell-specific alpha peaks when comparing alpha and beta cells, suggesting that they directly drive gene expression or repression in alpha cells by binding to uniquely accessible chromatin [4, 77]. For Irx2, this indicates that it directly drives gene expression or repression in alpha cells. For Insm1, which is expressed more uniformly in all three endocrine cell types, the role that it plays at promoter-proximal accessible chromatin is more complex and cannot be as readily inferred.

Between alpha and delta cells, the DNA binding motif for Pbx3, a transcription factor implicated in driving *Sst* expression in delta cells, was found preferentially enriched in accessible chromatin associated with promoter-proximal peaks [84], while the DNA motif for *Atf2*, identified as an enriched alpha cell motif in a previous human study, was found to be preferentially enriched on open chromatin associated with intronic peaks [5]. Between beta and delta cells, Insm1 and Nkx6.1 had motifs enriched at intronic chromatin regions [77, 89], while Fev – recently identified in pancreatic islet development—and Atf3* –* linked to enhancer regions in ﻿EndoC-bH1 cells—were identified as preferential to chromatin associated with distal-intergenic regions via their DNA binding motifs [11, 90].

Utilizing the R package *epiRomics*, we were able to derive a set of 16,651 putative enhancer peaks. Of these regions, 16.7% of enhancers were shared between beta and alpha cells, and 17.8% were shared between beta and delta cells, as opposed to the 8.18% shared between alpha and delta cells. Of note, our approach identified previously identified intronic enhancers, such as the one located on *Slc30a8 that is* regulated in part via the binding of the transcription actor Pdx1 and a promoter-proximal enhancer region upstream of the Pdx1 that is associated with Insm1*,* Neurod1*,* and Foxa2 binding, also identified by our approach (Fig. 9D) [77]. One final example of an enhancer is situated on the first intron of *Slc2a2.* having unique chromatin accessibility to beta cells, coupled with multiple transcription factor binding sites, including Pdx1*,* MafA*,* and Nkx6.1, could possibly explain the expression of the gene in beta cells while it is near undetectable between alpha and delta cells (Fig-S9B). *Slc2a2* plays a necessary role glucose-stimulated insulin secretion [103], with a recent study identifying a downstream enhancer regulating *Slc2a2* requiring the co-occupancy of both MafA and Neurod1, but also noting that complex interactions occur beyond the scope of this distal region [104]*.*

One limitation of our approach was that we were constrained to using ChIP data that had been made available by our colleagues in the field. The substantial majority are transcription factors associated with beta cells, with the results reflective of this limitation. For instance, 10.1% of the enhancer regions called were unique to beta cells, whereas we were only able to identify 1.53–2.47% unique to either delta or alpha cells (Fig-2E-F; Fig-S2E; Fig-S8A-I). The over-representation of beta cell-specific enhancer regions is probably explained by the fact that ChIP data for alpha and delta cell-specific enhancers obtained from pure populations of primary alpha and delta cells does not exist. While the majority of the transcription factors here are associated with beta cells, these data are still informative as open chromatin in delta and alpha regions without beta-cell transcription factor binding may be areas regulated through other layers of epigenetics, such as methylation, or via alpha- or delta- specific transcription factors for which no ChIP data is currently available [94]. While further validation of these regions lays beyond the scope of this study, such information would be readily integrated in the future in the multi-omics resource we described here.

## Conclusion

Here we have established a comprehensive picture of chromatin accessibility between major islet endocrine cell types and present the novel chromatin landscape of delta cells. We identified differential chromatin accessibility at promoter-proximal regions in both alpha cells and delta cells, when compared to beta cells. This finding was in line with a previous study in human islets, and further builds on previous literature in the field suggesting that both alpha and delta cells can transdifferentiate into beta cells. We also identified preferentially binding pattern differences across the annotated genome in transcription factor DNA-motifs across differentially enriched chromatin. Our evaluation of whether chromatin enrichment at the gene body is always correlated with gene expression enrichment also demonstrated that transcriptional regulation plays a role in determining cell fate rather than chromatin dynamics alone. Lastly, we devised and provided a simple approach to utilize and integrate a subset of these epigenomic datasets – ChIP and histone—alongside our ATAC-Seq chromatin data integrated with our previously published transcriptome of the FACS-purified alpha, beta, and delta cells through the development of an R package, *epiRomics*. This allowed for the visualization of integrated epigenomic data, and furthermore applies a novel approach to identify putative enhancer regions, enabling a high-resolution overview of key regions that may be responsible for driving cell fate decisions in pancreatic islet cell types. We have made this an interactive resource publicly available at https://www.huisinglab.com/epiRomics_2021/index.html. We believe our data and the tool we developed to visualize them to be a valuable resource to our field in pursuit of a full understanding of the epigenetic control over islet gene expression.

## Supplementary Information

**Additional file 1: Dataset-S1.** Annotated consensus chromatin peak set across alpha, beta and delta cells, along with differential enrichment results between the three pairwise comparisons.

**Additional file 2: Dataset-S2.** Congruent and incongruent genes of differentially expressed genes between the three pairwise comparisons. Congruent genes showed gene expression in the same direction as chromatin accessibility enrichment, whereas incongruent genes had opposing expression and enrichment.

**Additional file 3: Dataset-S3.** Unfiltered putative enhancer calls defined by open chromatin region in at least one of three cell types, overlapping the histone markers H3K27ac and H3K4me1.

**Additional file 4: Dataset-S4.** Filtered putative enhancer calls defined by open chromatin region in at least one of three cell types, overlapping the histone markers H3K27ac and H3K4me1.

**Additional file 5: Supplemental Figure 1.** FACS sorting gates used to isolate alpha, beta, and delta cells through our mouse reporter lines.

**Additional file 6: Supplemental Figure 2.** Validating more chromatin accessibility ATAC Seq and companion RNA-Seq expression in alpha, beta, and delta cells against hallmark genes governing its respective cell’s identity.

**Additional file 7: Supplemental Figure 3.** Chromatin enrichment does not always correlate with associated gene expression.

**Additional file 8: Supplemental Figure 4.** Evaluating KEGG and gene network enrichment (Alpha versus Beta).

**Additional file 9: Supplemental Figure 5.** Evaluating KEGG and gene network enrichment (Alpha versus Delta).

**Additional file 10: Supplemental Figure 6.** Evaluating KEGG and gene network enrichment (Beta versus Delta).

**Additional file 11: Supplemental Figure 7. **Transcription factor binding sites and histone mark overlap.

**Additional file 12: Supplemental Figure 8.** Aggregated transcription factor ATAC Seq and companion RNA-Seq expression in alpha, beta, and delta cells.

**Additional file 13: Supplemental Figure 9.** Further illustration of enhancer calls.

**Additional file 14: Supplemental Table 1.** Quality control metrics across all ATAC-Seq replicates.

**Additional file 15: Supplemental Table 2.** Aggregated dataset description and reference. A: Pancreatic islet ChIP Seq transcription factor data aggregated to identify enhancer and enhancer regions. B: Pancreatic islet histone data aggregated to identify enhancer and enhancer regions. The final approach utilized two histone marks deemed most relevant at delineating putative enhancer regions while taking into account a risk of both false positives and false negatives.

**Additional file 16: Supplemental Table 3.** Validating open chromatin peaks against known pancreatic islet ChIP binding sites. A: Evaluating the extent of open chromatin– as defined by our ATAC-Seq consensus peak set – contained binding sites for known, pancreatic islet transcription factors. Percent of open chromatin with associated binding sites ranged from 0.31-29.07%. The transcription factors Foxa2, Insm1, and Neurod1 had the highest number of binding sites. B: Evaluating the extent of each ChIP-Seq experiment’s binding site calls overlapped with open chromatin. Percent of overlap ranged from 0.35-63.79%. Nkx2.2, Neurod1, and Insm1 had the greatest overlap.

**Additional file 17: Supplemental Table 4.** Validating motif-calling approach against known ChIP binding sites. A: Pancreatic islet ChIP Seq transcription factor peak calls analyzed by the motif-calling method to determine sensitivity and specificity. True positive calls ranged from 0.59-57%, and false positives ranged from 1.19-8.34%. B: Pancreatic islet ChIP Seq transcription factor peak calls limited to open chromatin determined by the consensus peak set analyzed by the motif-calling method to determine sensitivity and specificity. True positive calls ranged from 4.71-65.10%, and false positives ranged from 1.68-8.81%.

## Data Availability

The datasets generated and analyzed for the current study are publicly available through GEO Accession GSE213970: All data that was aggregated and/or not originally generated for this study is available at GEO Accessions: GSE88778, GSE980673, or can be accessed through publicly available accessions listed in Table-S2.

## Abbreviations

ATAC-Seq: Assay for transposase-accessible chromatin using high throughput sequencing
Bulk RNA-Seq: High throughput sequencing at bulk resolution (*e.g.*, not single cell)
ChIP-Seq: Chromatin immunoprecipitation high throughput sequencing
FANTOM: Functional Annotation of the Mouse/Mammalian Genome
FRiP: Fraction of reads in peaks
KEGG: Kyoto Encyclopedia of Genes and Genomes
RNA-Seq: RNA high throughput sequencing
scATACSeq: Single-cell ATAC-Seq
TF: Transcription factor
TSS: Transcription start site
