# Supplementary material for: Chromatin accessibility differences between alpha, beta, and delta cells identifies common and cell type-specific enhancers
Source: BMC Genomics. 2023 Apr 17;24:202. doi: 10.1186/s12864-023-09293-6 (PMC10108528; doi:10.1186/s12864-023-09293-6)
Supplement: Supplementary file 1 — Additional file 1: Dataset-S1. Annotated consensus chromatin peak set across alpha, beta and delta cells, along with differential enrichment results between the three pairwise comparisons. [file 12864_2023_9293_MOESM1_ESM.pdf]

Supplemental Figure 6 – Evaluating KEGG and gene network enrichment (Beta versus Delta).

A

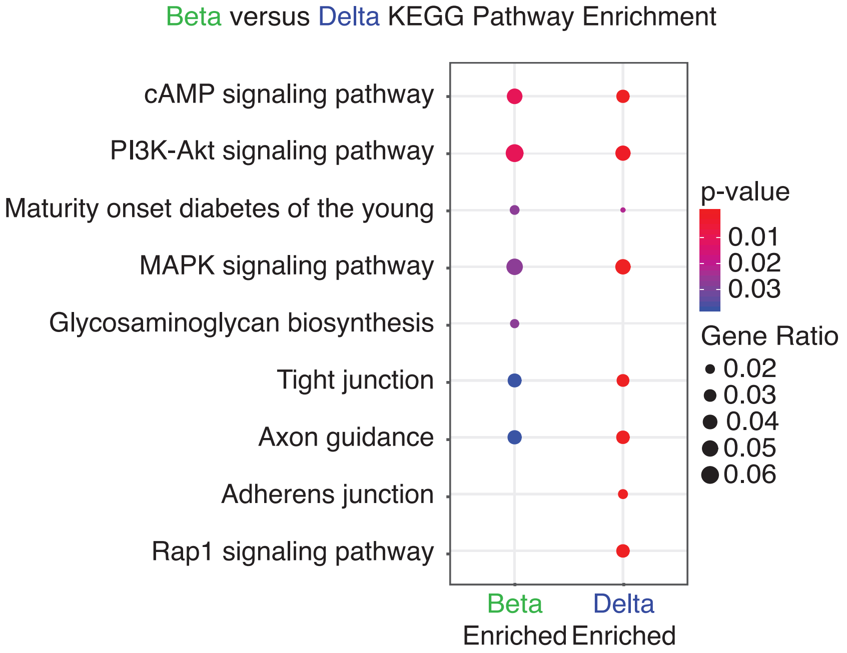

B

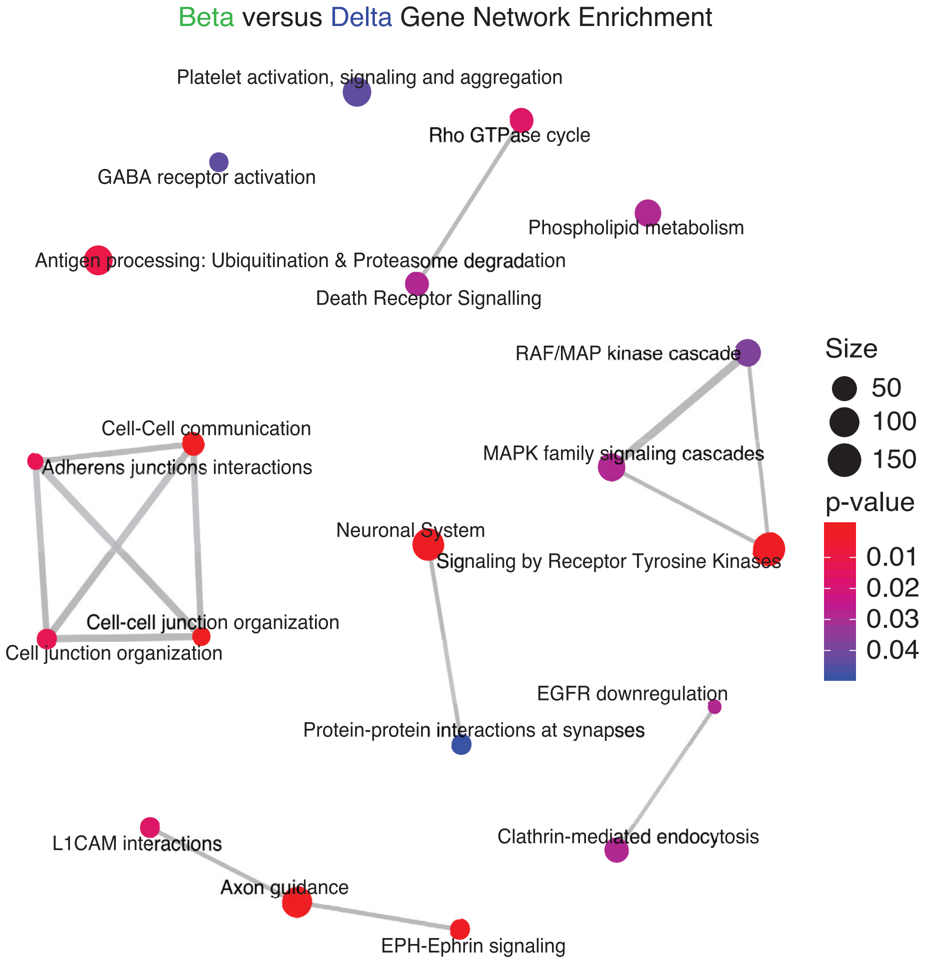

Fig-S6 – Evaluating KEGG and gene network enrichment across differentially enriched peaks between beta and delta cells. A: KEGG enrichment of differentially enriched peaks identified pathways common between the two cell types, or unique to one. B: Gene network enrichment indicative of possible functions of differentially enriched chromatin regions between the two cell types.
