# Supplementary material for: Chromatin accessibility differences between alpha, beta, and delta cells identifies common and cell type-specific enhancers
Source: BMC Genomics. 2023 Apr 17;24:202. doi: 10.1186/s12864-023-09293-6 (PMC10108528; doi:10.1186/s12864-023-09293-6)
Supplement: Supplementary file 2 — Additional file 2: Dataset-S2. Congruent and incongruent genes of differentially expressed genes between the three pairwise comparisons. Congruent genes showed gene expression in the same direction as chromatin accessibility enrichment, whereas incongruent genes had opposing expression and enrichment. [file 12864_2023_9293_MOESM2_ESM.pdf]

Supplemental Figure 7- Transcription factor binding sites and histone mark overlap.

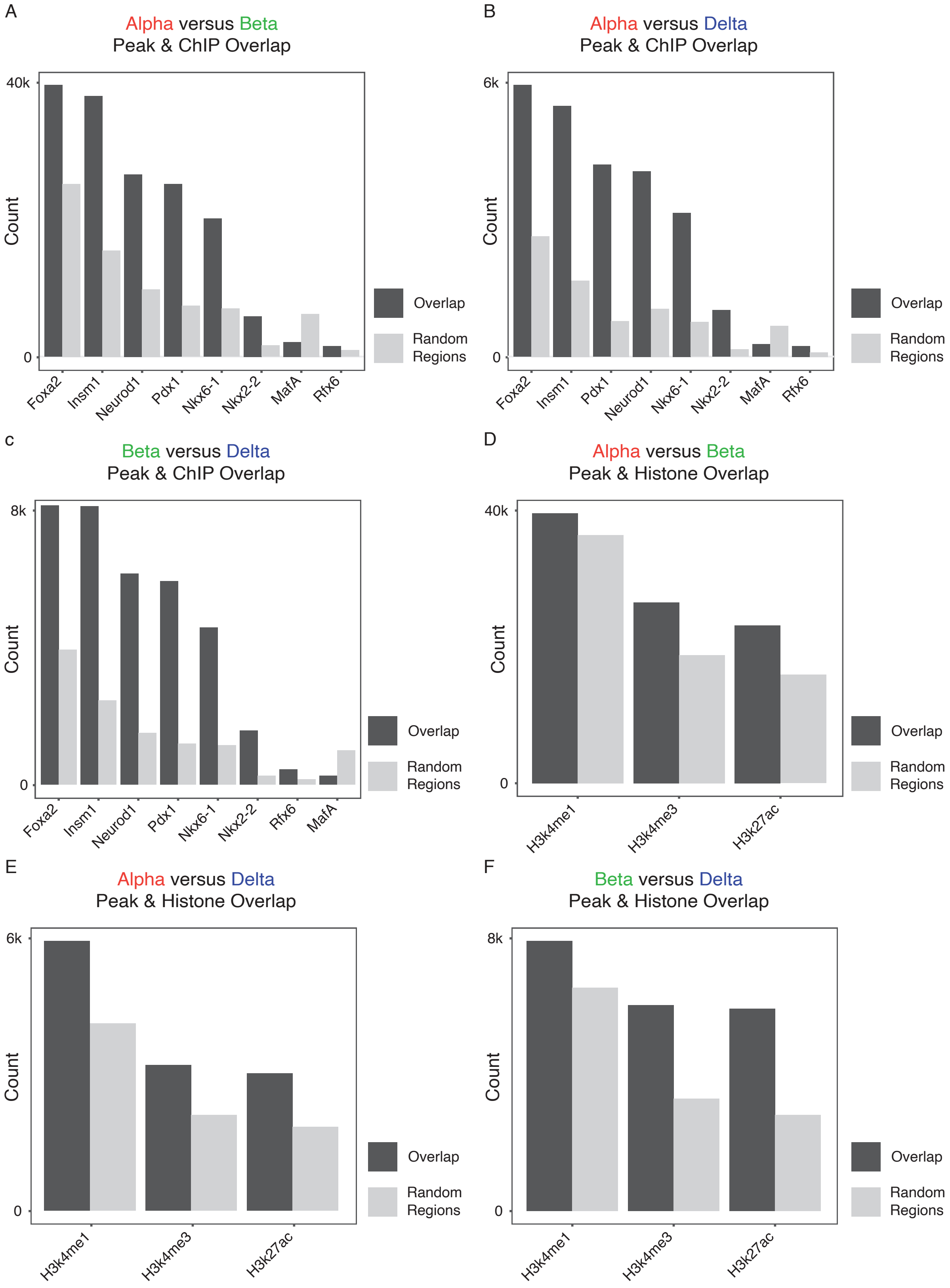

Fig-S7 – Verifying transcription factor binding sites and histone mark occurrence at chromatin peaks to determine significance (observed versus expected). A-C: Transcription factors on chromatin regions deemed enriched between differentially enriched chromatin across all three pairwise comparisons. The majority of transcription factors used in our analysis were deemed statistically significant when observed compared to predicted. D-F: Histone mark occurrence on chromatin regions deemed enriched between differentially enriched chromatin across all three pairwise comparisons. All histone marks used in our analysis were deemed statistically significant when observed compared to predicted.
