# Supplementary material for: Chromatin accessibility differences between alpha, beta, and delta cells identifies common and cell type-specific enhancers
Source: BMC Genomics. 2023 Apr 17;24:202. doi: 10.1186/s12864-023-09293-6 (PMC10108528; doi:10.1186/s12864-023-09293-6)
Supplement: Supplementary file 3 — Additional file 3: Dataset-S3. Unfiltered putative enhancer calls defined by open chromatin region in at least one of three cell types, overlapping the histone markers H3K27ac and H3K4me1. [file 12864_2023_9293_MOESM3_ESM.pdf]

Supplemental Figure 8 - Aggregated transcription factor ATAC Seq and companion RNA-Seq expression in alpha, beta, and delta cells.

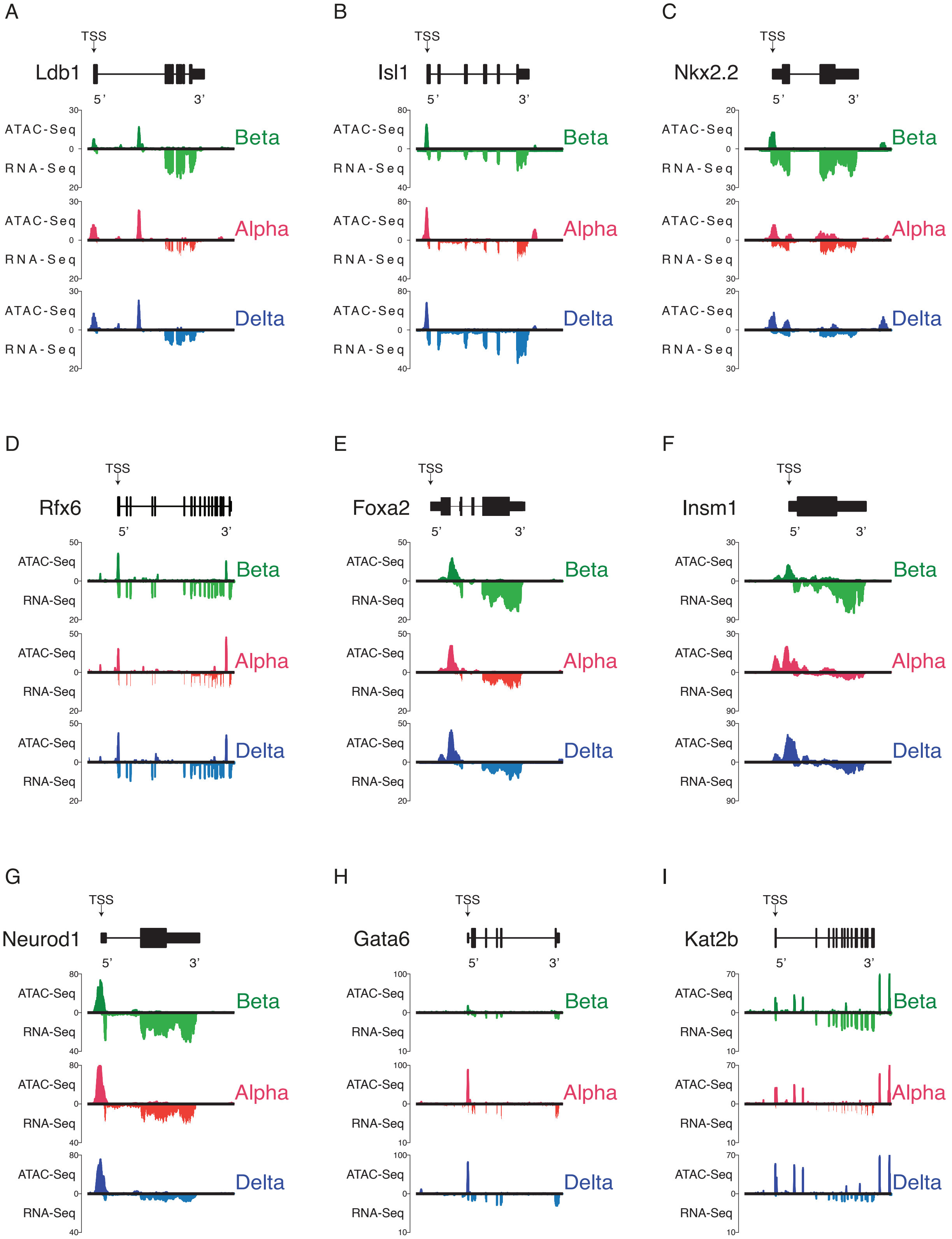

Fig-S8 – Aggregated transcription factor ATAC Seq and companion RNA-Seq expression in alpha, beta, and delta cells. All genes are oriented for 5' to 3' end. A-I: Chromatin accessibility and gene expression for aggregated ChIP datasets.
