# Supplementary material for: Chromatin accessibility differences between alpha, beta, and delta cells identifies common and cell type-specific enhancers
Source: BMC Genomics. 2023 Apr 17;24:202. doi: 10.1186/s12864-023-09293-6 (PMC10108528; doi:10.1186/s12864-023-09293-6)
Supplement: Supplementary file 4 — Additional file 4: Dataset-S4. Filtered putative enhancer calls defined by open chromatin region in at least one of three cell types, overlapping the histone markers H3K27ac and H3K4me1. [file 12864_2023_9293_MOESM4_ESM.pdf]

Supplemental Figure 9 – Further illustration of enhancer calls.

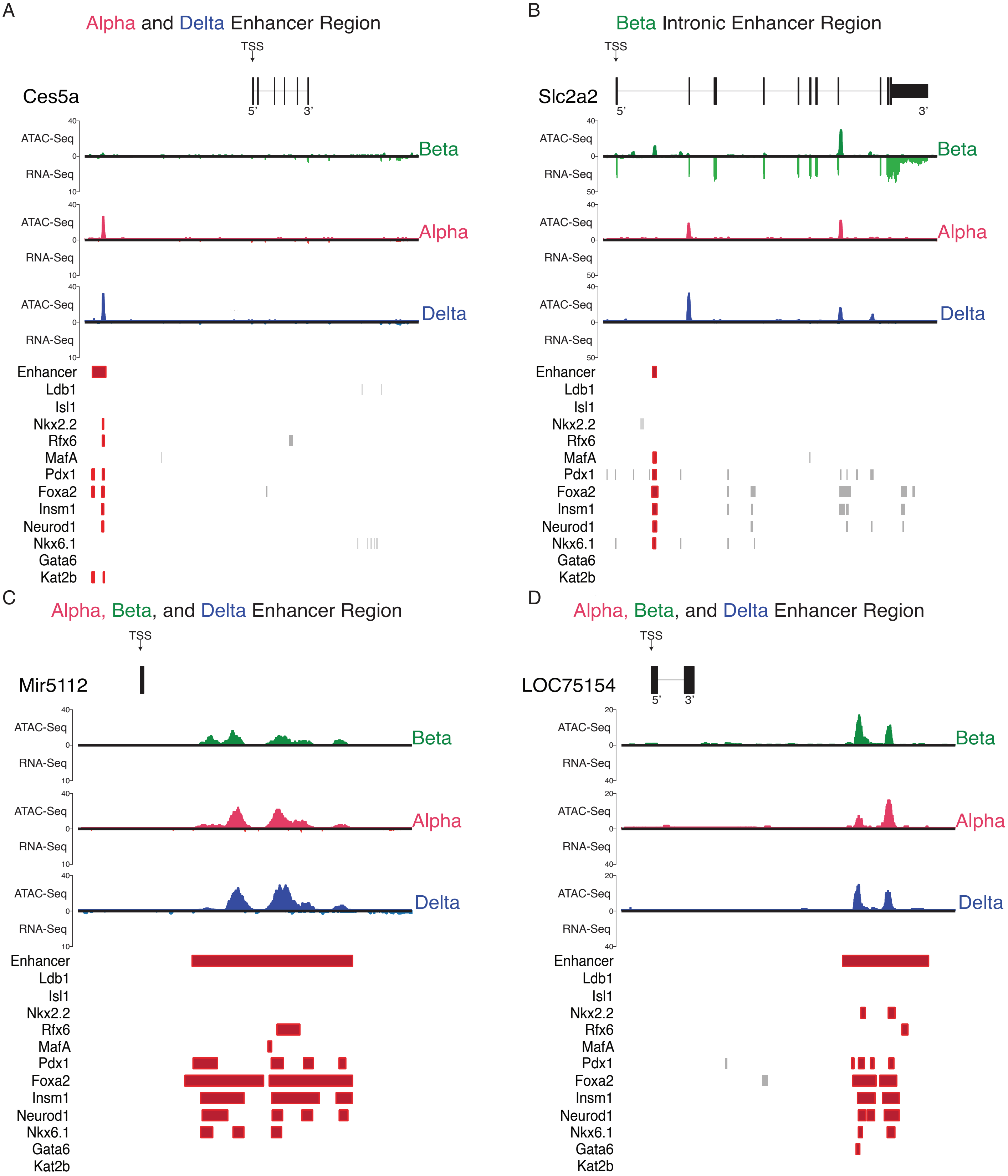

Fig-S9 – Further illustration of enhancer calls. A: Visualizing a common alpha and delta enhancer region, unavailable in beta cells. B: Further illustration of a beta-unique enhancer region, occurring on the first intron of *Slc2a2*, with 6 co-binding sites for multiple transcription actors. C-D: Two examples of called enhancer regions common across all three cell types. Both are in distal-intergenic regions of the genome and exhibit high transcription factor co-binding activity.
