# Supplementary material for: Chromatin accessibility differences between alpha, beta, and delta cells identifies common and cell type-specific enhancers
Source: BMC Genomics. 2023 Apr 17;24:202. doi: 10.1186/s12864-023-09293-6 (PMC10108528; doi:10.1186/s12864-023-09293-6)
Supplement: Supplementary file 5 — Additional file 5: Supplemental Figure 1. FACS sorting gates used to isolate alpha, beta, and delta cells through our mouse reporter lines. [file 12864_2023_9293_MOESM5_ESM.pdf]

Supplemental Table 1 - Quality control metrics across all ATAC-Seq replicates

Sample	# Cells	Sex	Library Prep	Library Type	Library Size (bp)	Total Reads	Total Reads Mapped	Unique Reads Mapped	FRiP
alpha 1	20000	F	NexteraDNA	single-end	444	28.1 M	18.5 M	2.5 M	0.37
alpha 2	20000	M	NexteraDNA	single-end	475	27.6 M	19.8 M	3.0 M	0.41
beta 1	20000	M	NexteraDNA	single-end	469	31.9 M	21.9 M	0.9 M	0.42
beta 2	20000	F	NexteraDNA	single-end	~450	36.1 M	24.8 M	1.3 M	0.42
beta 3	20000	M	NexteraDNA	single-end	~450	40.2 M	27.6 M	1.9 M	0.35
beta 4	20000	F	NexteraDNA	single-end	457	32.4 M	23.2 M	3.4 M	0.11
delta 1	20000	F	NexteraDNA	single-end	~450	42 M	29.9 M	3.2 M	0.42
delta 2	13500	M	NexteraDNA	single-end	~450	35.4 M	24.1 M	1.5 M	0.55
