# Supplementary material for: Chromatin accessibility differences between alpha, beta, and delta cells identifies common and cell type-specific enhancers
Source: BMC Genomics. 2023 Apr 17;24:202. doi: 10.1186/s12864-023-09293-6 (PMC10108528; doi:10.1186/s12864-023-09293-6)
Supplement: Supplementary file 6 — Additional file 6: Supplemental Figure 2. Validating more chromatin accessibility ATAC Seq and companion RNA-Seq expression in alpha, beta, and delta cells against hallmark genes governing its respective cell’s identity. [file 12864_2023_9293_MOESM6_ESM.pdf]

Supplemental Table 2 - Aggregated dataset description and reference. A: Pancreatic islet ChIP Seq transcription factor data aggregated to identify enhancer and enhancer regions. B: Pancreatic islet histone data aggregated to identify enhancer and enhancer regions. The final approach utilized two histone marks deemed most relevant at delineating putative enhancer regions while taking into account a risk of both false positives and false negatives.

A

**ChIP (Transcription Factor) Datasets**	
**Marker**	**Accession Info**
MafA	GSE30298
Nkx2.2	GSE79785
Rfx6	GSE62844
Neurod1	GSE54046
Foxa2	GSE54046
Isl1	GSE84759
Kat2b	GSE78860
Ldb1	GSE84759
Nkx6.1	GSE40975
Pdx1	E-MTAB-1143
Gata6	GSE57090
Insm1	GSE54046

B

**ChIP (Histone) Datasets**	
**Marker**	**Accession Info**
H3k27ac	GSE110648
H3k4me3	GSE110648
H3k4me1	GSE68618
H2ak119ub	GSE110648
H3k27me1	GSE110648
H3k36me3	GSE110648
H3K9me3	GSE110648
H3k9ac	GSE87530
H3k27me3	GSE110648
