# Supplementary material for: Chromatin accessibility differences between alpha, beta, and delta cells identifies common and cell type-specific enhancers
Source: BMC Genomics. 2023 Apr 17;24:202. doi: 10.1186/s12864-023-09293-6 (PMC10108528; doi:10.1186/s12864-023-09293-6)
Supplement: Supplementary file 7 — Additional file 7: Supplemental Figure 3. Chromatin enrichment does not always correlate with associated gene expression. [file 12864_2023_9293_MOESM7_ESM.pdf]

Supplemental Table 3 – Validating open chromatin peaks against known pancreatic islet ChIP binding sites. A: Evaluating the extent of open chromatin– as defined by our ATAC-Seq consensus peak set – contained binding sites for known, pancreatic islet transcription factors. Percent of open chromatin with associated binding sites ranged from 0.31-29.07%. The transcription factors Foxa2, Insm1, and Neurod1 had the highest number of binding sites. B: Evaluating the extent of each ChIP-Seq experiment's binding site calls overlapped with open chromatin. Percent of overlap ranged from 0.35-63.79%. Nkx2.2, Neurod1, and Insm1 had the greatest overlap.

A

**Percent of ATAC Peaks Overlapping with Transcription Factor Binding Sites**	
**Transcription Factor**	**Percent Overlap**
Foxa2	29.07%
Insm1	28.40%
Neurod1	20.09%
Pdx1	19.49%
Nkx6.1	15.44%
Nkx2.2	4.56%
MafA	1.56%
Rfx6	1.19%
Gata6	1.02%
Ldb1	0.41%
Kat2b	0.35%
Isl1	0.31%

B

**Percent of Transcription Factor Binding Sites Overlapping with ATAC Peaks**	
**Transcription Factor**	**Percent Overlap**
Nkx2.2	63.79%
Neurod1	55.07%
Insm1	51.49%
Pdx1	50.85%
Nkx6.1	43.56%
Gata6	40.20%
Rfx6	35.65%
Foxa2	34.86%
Isl1	9.95%
Ldb1	5.46%
MafA	3.27%
Kat2b	0.35%
