# Supplementary material for: Chromatin accessibility differences between alpha, beta, and delta cells identifies common and cell type-specific enhancers
Source: BMC Genomics. 2023 Apr 17;24:202. doi: 10.1186/s12864-023-09293-6 (PMC10108528; doi:10.1186/s12864-023-09293-6)
Supplement: Supplementary file 8 — Additional file 8: Supplemental Figure 4. Evaluating KEGG and gene network enrichment (Alpha versus Beta). [file 12864_2023_9293_MOESM8_ESM.pdf]

Supplemental Table 4 - Validating motif-calling approach against known ChIP binding sites. A: Pancreatic islet ChIP Seq transcription factor peak calls analyzed by the motif-calling method to determine sensitivity and specificity. True positive calls ranged from 0.59-57%, and false positives ranged from 1.19-8.34%. B: Pancreatic islet ChIP Seq transcription factor peak calls limited to open chromatin determined by the consensus peak set analyzed by the motif-calling method to determine sensitivity and specificity. True positive calls ranged from 4.71-65.10%, and false positives ranged from 1.68-8.81%.

A

**Transcription Factor Motif Calls against ChIP Peaks Validation**				
**Transcription Factor**	**False Negative**	**True Positive**	**False Positive**	**True Negative**
Rfx6	43.00%	57%	8.34%	91.66%
Gata6	77.58%	22.42%	1.78%	98.22%
Foxa2	78.17%	21.83%	6.38%	93.62%
Nkx6.1	82.05%	17.95%	6.89%	93.11%
Nkx2.2	89.48%	10.52%	2.98%	97.02%
Insm1	93.39%	6.61%	3.63%	96.37%
Pdx1	95.51%	4.49%	1.26%	98.74%
Isl1	98.33%	1.67%	0.51%	99.49%
MafA	99.41%	0.59%	1.19%	98.81%

B

**Transcription Factor Motif Calls against ChIP Peaks (Open ATAC only) Validation**				
**Transcription Factor**	**False Negative**	**True Positive**	**False Positive**	**True Negative**
Rfx6	34.90%	65.10%	8.81%	91.19%
Foxa2	80.11%	19.89%	8.92%	91.08%
Gata6	83.80%	16.20%	1.98%	98.02%
Nkx6.1	88.61%	11.39%	8.74%	91.26%
Nkx2.2	90.28%	9.72%	3.69%	96.31%
MafA	91.29%	8.71%	1.73%	98.27%
Insm1	91.74%	8.26%	3.97%	96.03%
Isl1	94.12%	5.88%	0.74%	99.26%
Pdx1	95.30%	4.71%	1.68%	98.32%
