# Supplementary material for: Chromatin accessibility differences between alpha, beta, and delta cells identifies common and cell type-specific enhancers
Source: BMC Genomics. 2023 Apr 17;24:202. doi: 10.1186/s12864-023-09293-6 (PMC10108528; doi:10.1186/s12864-023-09293-6)
Supplement: Supplementary file 13 — Additional file 13: Supplemental Figure 9. Further illustration of enhancer calls. [file 12864_2023_9293_MOESM13_ESM.pdf]

Supplemental Figure 1 - FACS sorting gates used to isolate alpha, beta, and delta cells through our mouse reporter lines.

A

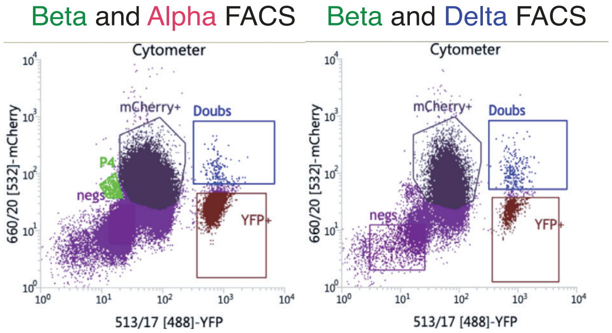

Fig-S1 – FACS sorting gates used to isolate alpha, beta, and delta cells through our mouse reporter lines. FACS sorting gates isolating beta cells (Ins2-mCherry+) from either alpha (Gcg-YFP+) or delta cells (Sst-YFP+). Double negatives are non-beta and non-alpha or non-delta cells. Double positives (mCherry/YFP+) represent cells with both Ins2 expression and Gcg or Sst expression, reflective of transdifferentiated beta cells. These were not included in any of the samples.
