# Supplementary material for: Chromatin accessibility differences between alpha, beta, and delta cells identifies common and cell type-specific enhancers
Source: BMC Genomics. 2023 Apr 17;24:202. doi: 10.1186/s12864-023-09293-6 (PMC10108528; doi:10.1186/s12864-023-09293-6)
Supplement: Supplementary file 14 — Additional file 14: Supplemental Table 1. Quality control metrics across all ATAC-Seq replicates. [file 12864_2023_9293_MOESM14_ESM.pdf]

Supplemental Figure 2 – Validating more chromatin accessibility ATAC Seq and companion RNA-Seq expression in alpha, beta, and delta cells against hallmark genes governing its respective cell's identity.

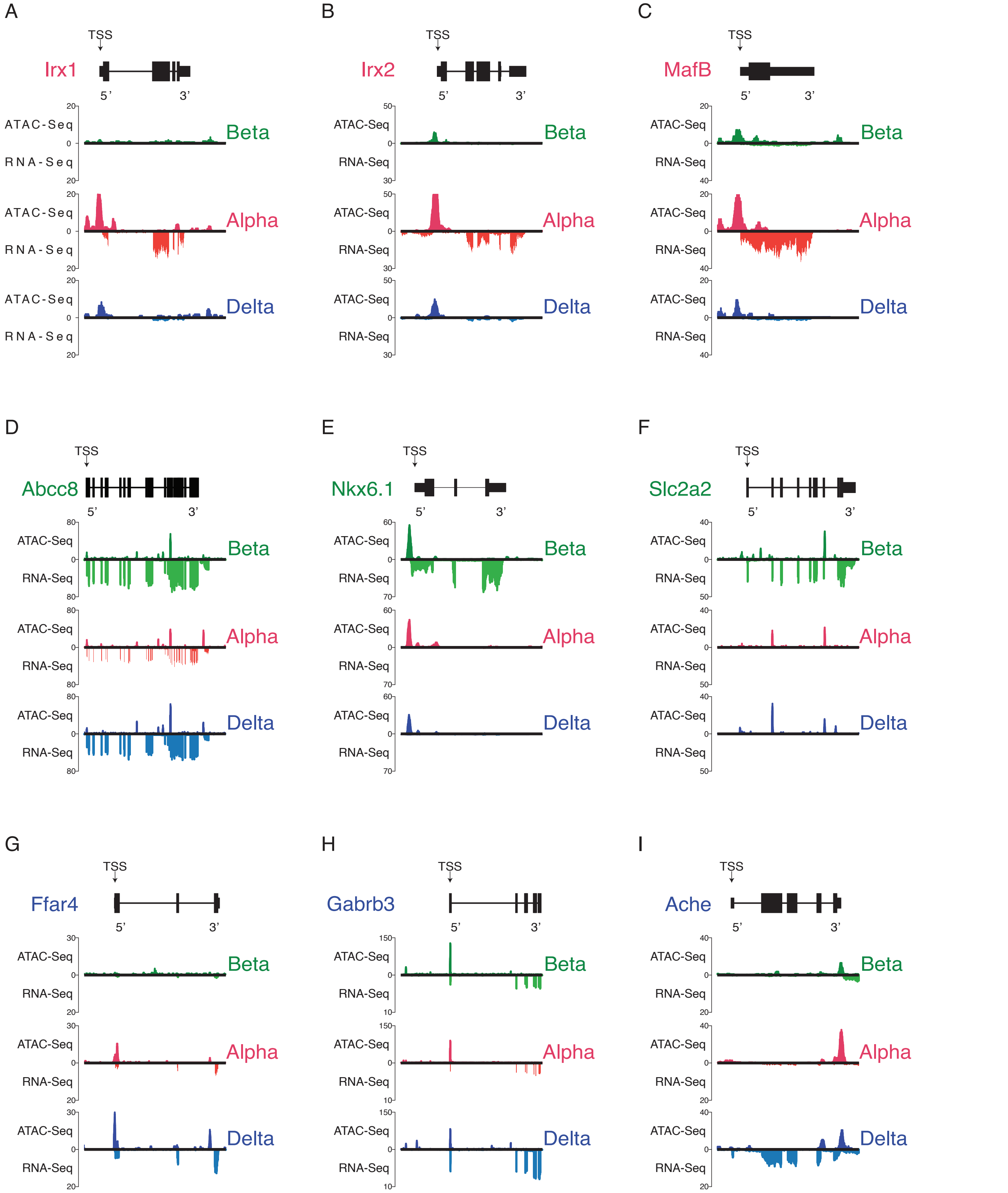

Fig-S2 – Validating more chromatin accessibility ATAC Seq and companion RNA-Seq expression in alpha, beta, and delta cells against hallmark genes governing its respective cell's identity. All genes are oriented for 5' to 3' end. A-C: Normalized chromatin accessibility and transcript expression across alpha cell hallmark genes *Irx1*, *Irx2*, and *MafB*. D-F: Normalized chromatin accessibility and transcript expression across beta cell hallmark genes *Abcc8*, *Nkx6.1*, and *Slc2a2*. G-I: Normalized chromatin accessibility and transcript expression across delta cell hallmark genes *Ffar4*, *Gabrb3*, and *Ache*.
