# Supplementary material for: Chromatin accessibility differences between alpha, beta, and delta cells identifies common and cell type-specific enhancers
Source: BMC Genomics. 2023 Apr 17;24:202. doi: 10.1186/s12864-023-09293-6 (PMC10108528; doi:10.1186/s12864-023-09293-6)
Supplement: Supplementary file 15 — Additional file 15: Supplemental Table 2. Aggregated dataset description and reference. A: Pancreatic islet ChIP Seq transcription factor data aggregated to identify enhancer and enhancer regions. B: Pancreatic islet histone data aggregated to identify enhancer and enhancer regions. The final approach utilized two histone marks deemed most relevant at delineating putative enhancer regions while taking into account a risk of both false positives and false negatives. [file 12864_2023_9293_MOESM15_ESM.pdf]

Supplemental Figure 3 - Chromatin enrichment does not always correlate with associated gene expression.

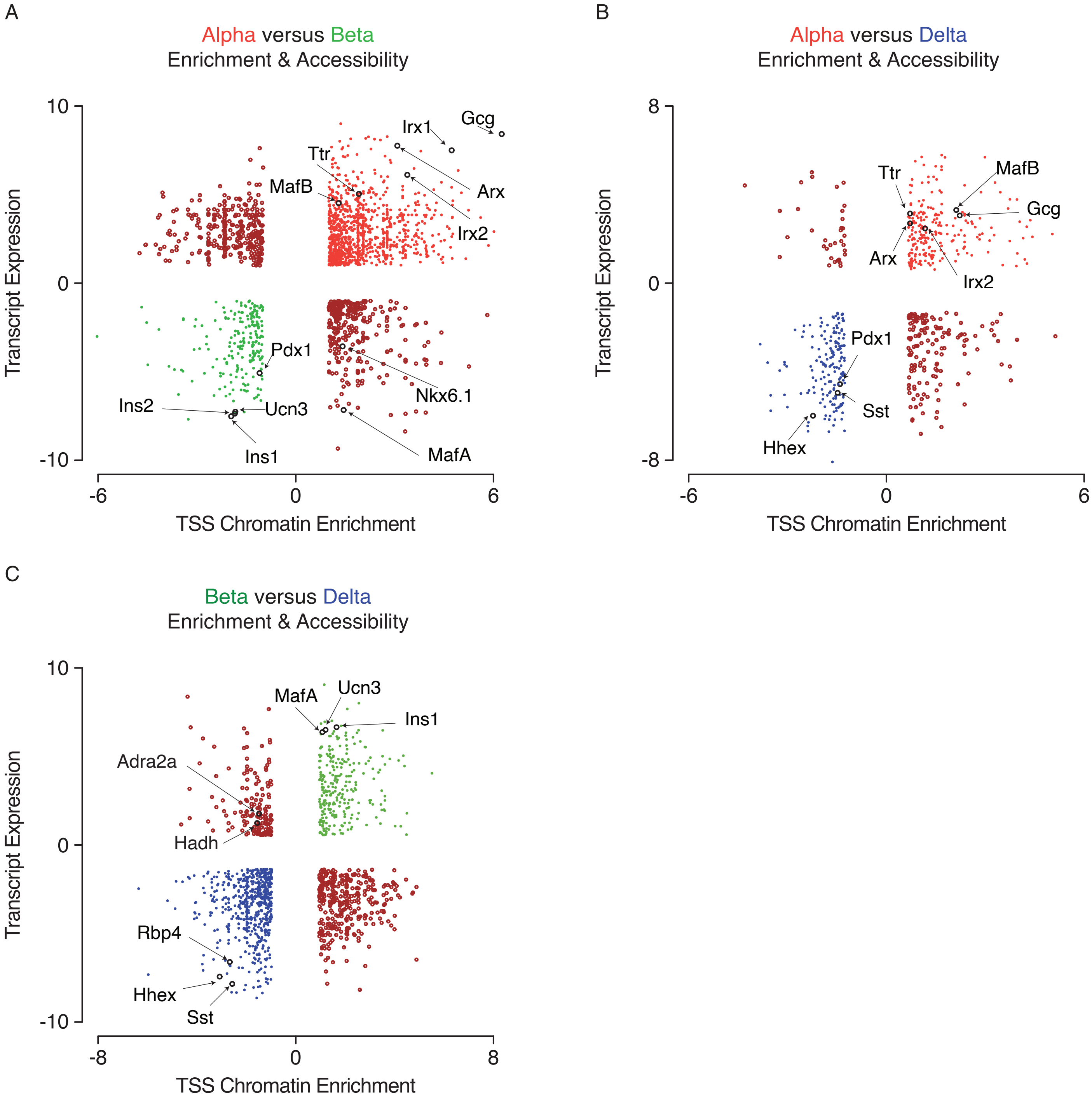

Fig-S3 – Chromatin enrichment does not always correlate with associated gene expression. Select hallmark genes defining demonstrating congruent and incongruent chromatin and gene enrichment for cell-specific markers. A: Differentially enriched chromatin at TSS regions and respective gene expression between alpha and beta cells. The majority of cell-specific markers show TSS-enrichment within the cell type of expression. Notably, *Nkx6.1* and *MafA* show TSS enrichment in alpha cells, despite being transcription factors associated with beta cells. B: Differentially enriched chromatin at TSS regions and respective gene expression between alpha and delta cells. C: Differentially enriched chromatin at TSS regions and respective gene expression between beta and beta cells. The majority of cell-specific markers show TSS-enrichment within the cell type of expression.
