# Supplementary material for: Chromatin accessibility differences between alpha, beta, and delta cells identifies common and cell type-specific enhancers
Source: BMC Genomics. 2023 Apr 17;24:202. doi: 10.1186/s12864-023-09293-6 (PMC10108528; doi:10.1186/s12864-023-09293-6)
Supplement: Supplementary file 16 — Additional file 16: Supplemental Table 3. Validating open chromatin peaks against known pancreatic islet ChIP binding sites. A: Evaluating the extent of open chromatin– as defined by our ATAC-Seq consensus peak set – contained binding sites for known, pancreatic islet transcription factors. Percent of open chromatin with associated binding sites ranged from 0.31-29.07%. The transcription factors Foxa2, Insm1, and Neurod1 had the highest number of binding sites. B: Evaluating the extent of each ChIP-Seq experiment’s binding site calls overlapped with open chromatin. Percent of overlap ranged from 0.35-63.79%. Nkx2.2, Neurod1, and Insm1 had the greatest overlap. [file 12864_2023_9293_MOESM16_ESM.pdf]

Supplemental Figure 4 – Evaluating KEGG and gene network enrichment (Alpha versus Beta).

A

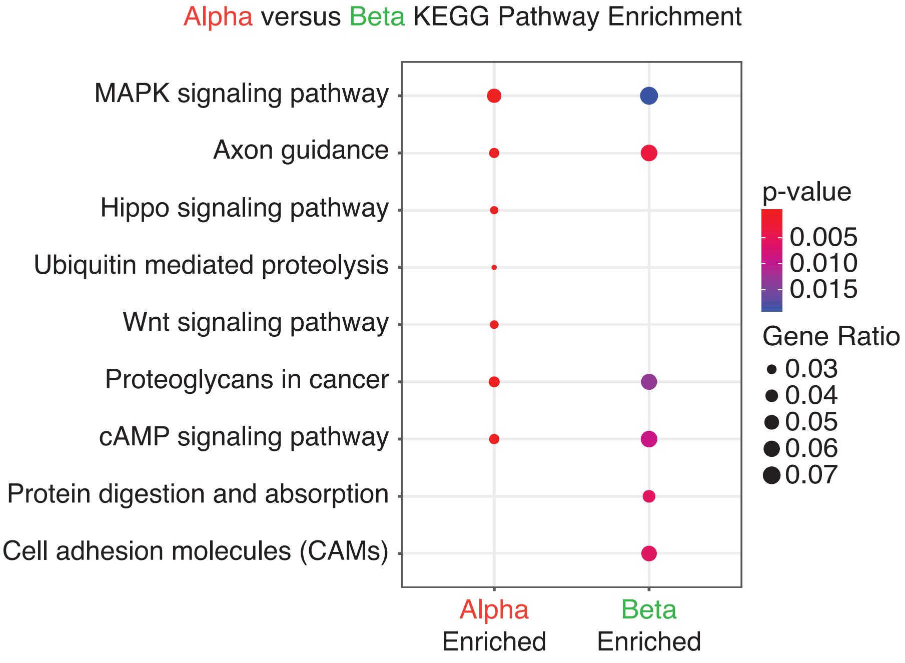

B

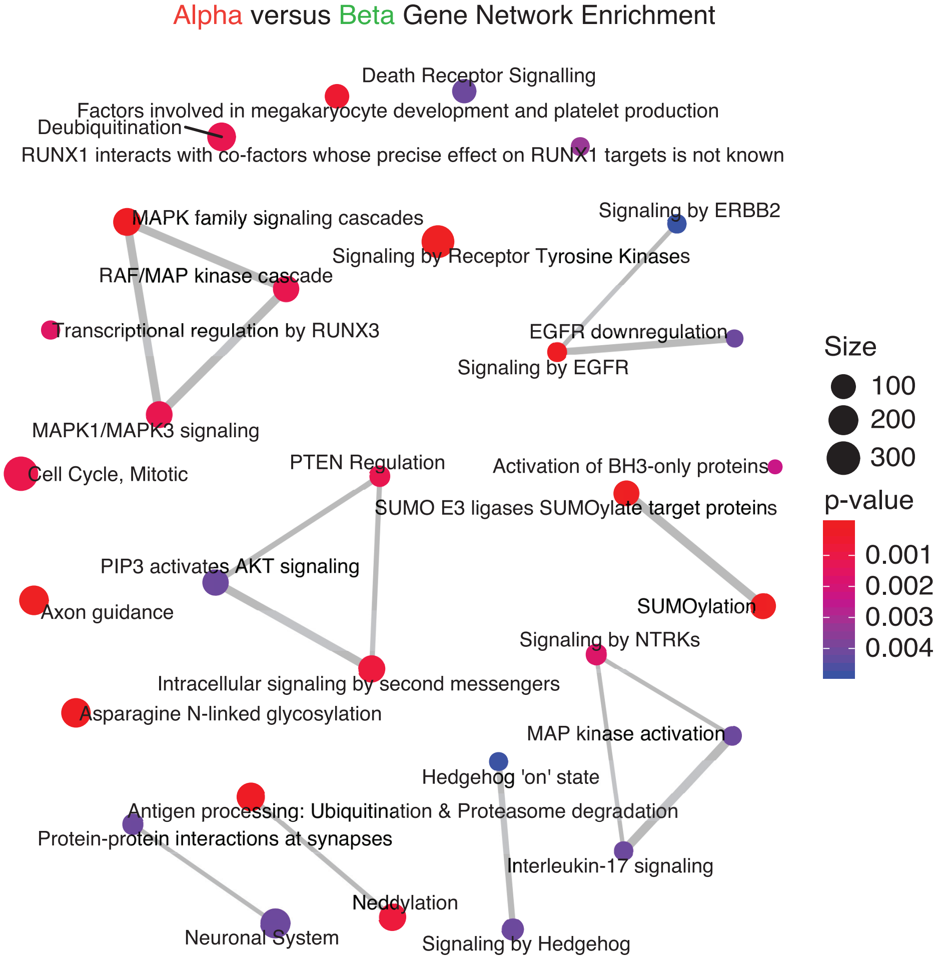

Fig-S4 – Evaluating KEGG and gene network enrichment across differentially enriched peaks between alpha and beta cells. A: KEGG enrichment of differentially enriched peaks identified pathways common between the two cell types, or unique to one. B: Gene network enrichment indicative of possible functions of differentially enriched chromatin regions between the two cell types.
