# Supplementary material for: Chromatin accessibility differences between alpha, beta, and delta cells identifies common and cell type-specific enhancers
Source: BMC Genomics. 2023 Apr 17;24:202. doi: 10.1186/s12864-023-09293-6 (PMC10108528; doi:10.1186/s12864-023-09293-6)
Supplement: Supplementary file 17 — Additional file 17: Supplemental Table 4. Validating motif-calling approach against known ChIP binding sites. A: Pancreatic islet ChIP Seq transcription factor peak calls analyzed by the motif-calling method to determine sensitivity and specificity. True positive calls ranged from 0.59-57%, and false positives ranged from 1.19-8.34%. B: Pancreatic islet ChIP Seq transcription factor peak calls limited to open chromatin determined by the consensus peak set analyzed by the motif-calling method to determine sensitivity and specificity. True positive calls ranged from 4.71-65.10%, and false positives ranged from 1.68-8.81%. [file 12864_2023_9293_MOESM17_ESM.pdf]

Supplemental Figure 5 - Evaluating KEGG and gene network enrichment (Alpha versus Delta).

A

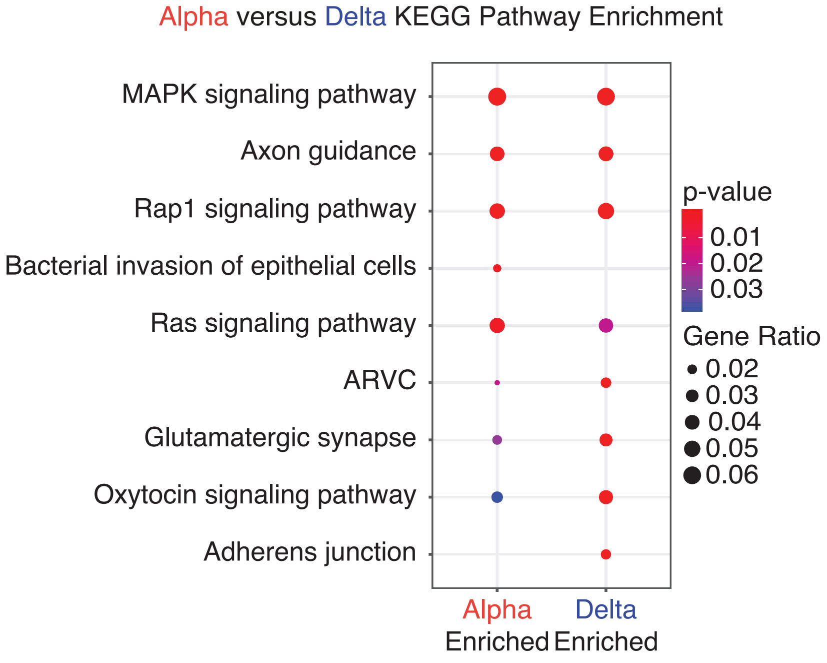

B

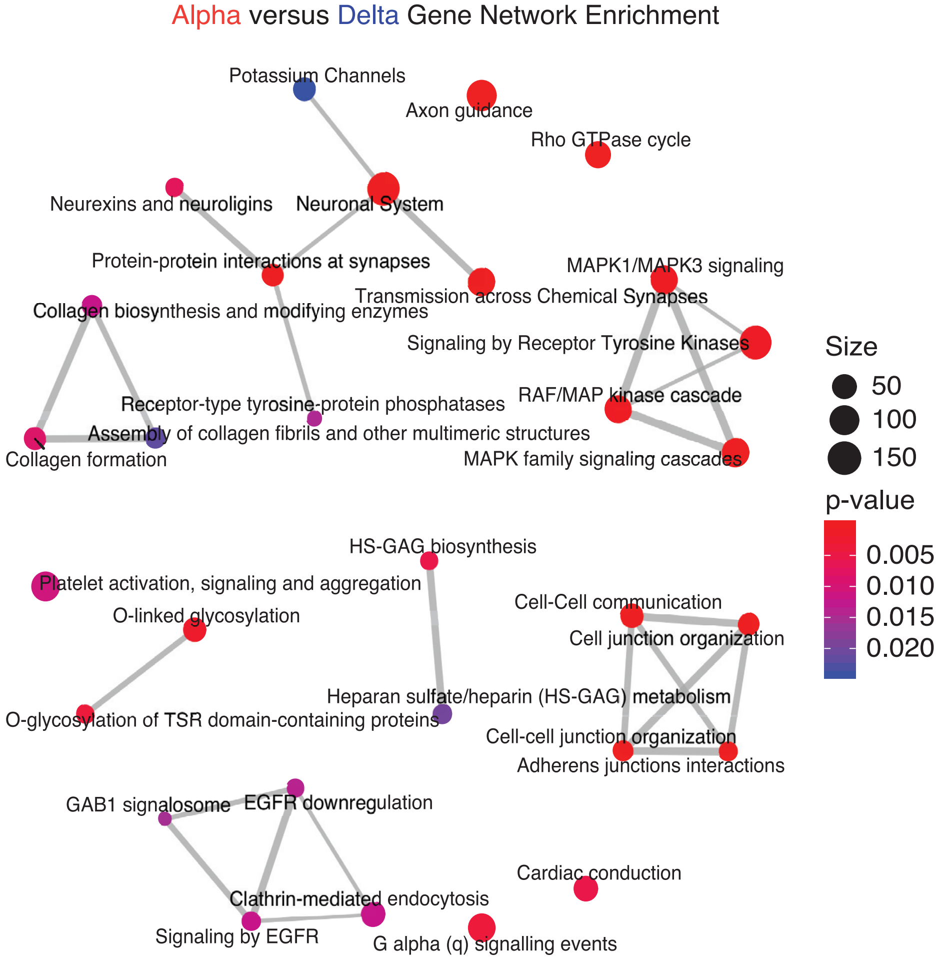

Fig-S5 – Evaluating KEGG and gene network enrichment across differentially enriched peaks between alpha and delta cells. A: KEGG enrichment of differentially enriched peaks identified pathways common between the two cell types, or unique to one. B: Gene network enrichment indicative of possible functions of differentially enriched chromatin regions between the two cell types.
